# Modulation of growth, microcystin production, and algal-bacterial interactions of the bloom-forming algae *Microcystis aeruginosa* by a novel bacterium recovered from its phycosphere

**DOI:** 10.3389/fmicb.2024.1295696

**Published:** 2024-03-01

**Authors:** Yao Xiao, Mijia Du, Yang Deng, Qinglin Deng, Xin Wang, Yiwen Yang, Binghuo Zhang, Yu-Qin Zhang

**Affiliations:** ^1^College of Pharmacy and Life Science, Jiujiang University, Jiujiang, China; ^2^Institute of Medicinal Biotechnology, Chinese Academy of Medical Sciences & Peking Union Medical College, Beijing, China

**Keywords:** *Mucilaginibacter lacusdianchii*, harmful algal bloom, bacterial symbiont, genome, algal-bacterial interactions

## Abstract

Harmful algal blooms (HABs) in natural waters are of escalating global concern due to their detrimental impact on environmental health. Emerging evidence indicates that algae-bacteria symbionts can affect HAB features, though much about this interplay remains largely unexplored. The current study isolated a new species of *Mucilaginibacter* (type strain JXJ CY 39^T^) from culture biomass of the bloom-causing *Microcystis aeruginosa* FACHB-905 (Maf) from Lake Dianchi, China. Strain JXJ CY 39^T^ was an aerobic, Gram-stain-negative rod bacterium that grew at 5–38°C, pH 4.0–11.0, and 0–3.0% NaCl. Taxonomic evaluation proposed a new species, with *Mucilaginibacter lacusdianchii* sp. nov., as the species epithet. Experimental results revealed that strain JXJ CY 39^T^ spurred the growth of Maf by supplying soluble phosphorus and nitrogen during cultivation, despite the unavailability of soluble phosphorus and nitrogen. Additionally, by producing the plant hormone indole-3-acetate, strain JXJ CY 39^T^ possibly impacted Maf’s functionality. Results from co-culture experiments with other strains from Maf biomass showed possible effects of strain JXJ CY 39^T^ on the relationship between Maf and other cohabiting bacteria, as well as microcystin toxin production characteristics. Although Maf could foster the growth of strain JXJ CY 39^T^ by supplying organic carbon, the strain’s growth could be regulated via specific chemical compounds based on antibiotic assays. Community composition analysis disclosed that this *Mucilaginibacter* strain positively affected Maf’s growth and modified densities and types of bacteria linked to Maf. Overall, these results suggest that the interactions between important HAB-causing organisms and their attached bacteria are complex, dynamic, and may influence the growth characteristics of algae.

## Highlights

- Harmful algal blooms (HABs) are persistent environmental problems caused by algae.
- A novel bacterium (JXJ CY 39^T^) was isolated and characterized from the phycosphereof an HAB-alga (Maf).
- JXJ CY 39^T^ modulated the growth and microcystin production of Maf.
- JXJ CY 39^T^ also influenced the attached bacterial assemblages of Maf.
- Bacterial symbionts of HAB-algae should be more carefully considered in treatments.

## 1 Introduction

Various types of algae cause harmful algal blooms (HABs), which pose an accelerating concern in both oceans and freshwater worldwide owing to their expansion in recent times, primarily due to global climate changes such as warming trends (Glibert et al., 2005). These HABs impair water ecosystems considerably, including posing threats to environment and human health by producing potent toxins (Grattan et al., 2016).

Distinct exudates from different algal species exhibit different chemical characteristics that result in the coexistence of varying bacterial partners with specific algae (Yang et al., 2017), leading to extremely diverse and complex interactions (Shao et al., 2014) that can include nutrient exchange, signal transduction, and gene transfer (Kouzuma and Watanabe, 2015). Among these, nutrient exchange is considered the most common type of interactions (Kouzuma and Watanabe, 2015) and is frequently the basis of algal–bacterial mutualisms (Cooper and Smith, 2015). Specifically, algae provide attached bacteria with a safer habitat and protection from grazing, in addition to dissolved organic matter compounds like polysaccharides, while attached bacteria provide algae with various nutrients like bio-available P, N, CO_2_, microelements, and vitamins (Yang and Xiao, 2011). The mutualisms between algae and attached bacteria are consequently highly mediated by the provisioning of nutrients from bacteria that may be able to initiate and maintain symbiotic relationships (Cooper and Smith, 2015). Importantly, the various interactions that have co-evolved between algae and their attached bacteria (Kouzuma and Watanabe, 2015; Cirri and Pohnert, 2019) have significant, but distinct impacts on the occurrence, duration and decline of algal blooms (Shao et al., 2014; Zhang et al., 2019). Consequently, the importance of attached bacteria must be considered when identifying treatments to control and mitigate HABs.

*Microcystis aeruginosa* is a common group of bloom-inducing Cyanobacteria, leading source of hepatotoxic microcystins (MCs) specifically produced in freshwater (Dawson, 1998; Park et al., 2009). Among over 270 different MC compounds (Lin et al., 2021), MC-LR (contains leucine and arginine) is the most prevalent in both natural water blooms and *M. aeruginosa* cultures in laboratory (Vasconcelos et al., 1996; Liu et al., 2012). *M. aeruginosa* can also secrete extracellular mucilage primarily composed of polysaccharides that provide habitats for bacteria that attach to *Microcystis* colonies and interact with the algae (Dziallas and Grossart, 2011; Parveen et al., 2013). Many bacteria attached to *M. aeruginosa* extracellular matrices have been isolated and identified, including in the genera *Erythrobacter* (Zhao et al., 2011), *Gordonia, Burkholderia* (Zhao et al., 2012), *Sphingomonas* (Guo et al., 2020), *Pseudomonas* (Yang and Xiao, 2011), and numerous other taxa within the phyla *Actinobacteria*, *Bacteroidetes*, *Proteobacteria*, and *Deinococcus-Thermus* (Berg et al., 2009; Guo et al., 2020). Indeed, many of these bacterial taxa have evolved with their algal hosts (Ramanan et al., 2016).

In this study, a bacterium JXJ CY 39^T^ was purified from the attached bacterial community of algal *M. aeruginosa* FACHB-905 (Maf), which was previously obtained from Lake Dianchi. Lake Dianchi is the largest lake on the Yunnan-Guizhou plateau and is one of the most seriously pollution-impacted eutrophic freshwater lakes in China. Cyanobacterial blooms primarily dominated by *M. aeruginosa* have gradually became a common phenomenon in Lake Dianchi since the 1980’s due to increasing dumping of various wastes into the lake (Liu, 1999). Indeed, HABs occurred almost yearly from 1990 to 2010. Polyphasic taxonomic study revealed that JXJ CY 39^T^ represents a novel species of *Mucilaginibacter*, belonging to *Bacteroidota* which are the most common phylum attached to *Microcystis* HABs (Ndlela et al., 2018). The genus *Mucilaginibacter*, a large group in *Bacteroidota*, was first described by Pankratov et al. (2007) and later emended by Urai et al. (2008), Baik et al. (2010), and Chen et al. (2014). *Mucilaginibacter* currently comprises 80 species with validly published names^1^ (List of Prokaryotic names with Standing in Nomenclature; December 9, 2023). Here, the interactions between Maf and its attached bacteria, including strain JXJ CY 39^T^, were investigated with co-culture experiments to understand if and how Maf controls the growth of strain JXJ CY 39^T^ and vice-a-versa. Moreover, the bacterial communities associated with Maf that was co-cultured with and without strain JXJ CY 39^T^ were compared over 35 days with 16S rRNA gene amplicon sequence analysis. Taken together, these experiments were used to more comprehensively understand the interactions among Maf, JXJ CY 39^T^, and other Maf-attached bacteria. The new insights reported here is to inform the treatment of HABs via consideration of their commensal bacteria.

## 2 Materials and methods

### 2.1 Polyphasic taxonomy study on strain JXJ CY 39^T^

#### 2.1.1 Isolation of the microorganisms

Algal *Microcystis aeruginosa* FACHB-905 (Maf) was obtained from Lake Dianchi, China, and subsequently incubated in our lab as described previously (Zhang et al., 2016c). Bacteria, including strain JXJ CY 39^T^ and other eight attached strains, were isolated from the culture biomass of Maf using International Streptomyces Project 2 (ISP 2) agar medium according to the described procedure (Xiao et al., 2022a). Isolated bacteria were preserved at 4.0°C using ISP 2 slants and − 80.0°C using glycerol suspensions (30–50%, v/v). Then we tried to purify Maf on BG11 (Blue-Green Medium) agar plate (Allen, 1968) to get rid of the culturable bacteria attached to the Maf. The purified-Maf was confirmed when no bacterial colony was formed by inoculation of the Maf onto newly prepared ISP 2 agar and nutrient agar, respectively. Purified-Maf was cultured using BG11 liquid medium at 25.0°C under illumination of 30 μmol photon/m^2^/s with a 12 h: 12 h light: dark cycle. In the subsequent experiments, the purified-Maf culture-system without supplement with other attached bacteria served as a basal control group.

#### 2.1.2 Morphological and physiological characterization of strain JXJ CY 39^T^

Bacterial cellular morphology was observed with transmission electron microscopy (TEM; JEM-2100, JEOL) after growing on ISP 2 agar medium at 28.0°C for four days. Gram stains were conducted using standard procedures. Catalase activity was evaluated from bubble production after addition of a drop of 3% (*v*/*v*) H_2_O_2_ on cell biomass. Growth at different temperatures (5, 10, 15, 20, 26, 28, 30, 32, 35, 38, 41, and 45°C), pH (2.0–12.0 in 1.0 intervals), and NaCl concentrations (0–10.0% *w*/*v*, in 1% intervals) was conducted using ISP 2 agar medium as the basal growth medium. Hydrolysis of starch and Tween (20, 40, and 80) tests were performed according to methods described by Dong and Cai (2001). Other physiological and biochemical characteristics were determined using API systems.

#### 2.1.3 Chemotaxonomic characteristics

Cellular biomass subjected to chemical analysis was obtained from pure cultures grown on ISP 2 agar medium at 28.0°C for four days. Cellular fatty acids were analyzed using the MIDI System (Sherlock version 6.1; MIDI database: TSBA6). Polar lipids were extracted and analyzed by two-dimensional thin-layer chromatography (Minnikin et al., 1979). Further, respiratory quinones were extracted as described by Collins et al. (1977) and analyzed with HPLC (Tamaoka et al., 1983).

#### 2.1.4 Phylogenetic and genome sequence analysis

Genomic DNA was prepared by using Rapid Bacterial Genomic DNA Isolation Kit (Sangon Biotech, Shanghai, China), and subsequently, the 16S rRNA gene sequences were obtained as described previously (Zhang et al., 2016b), and compared against those in the EzBioCloud Database. Phylogenetic trees were reconstructed using neighbor-joining (Saitou and Nei, 1987), maximum-parsimony (Fitch, 1971), and Maximum-Likelihood (Felsenstein, 1981) algorithms in MEGA version 11 (Tamura et al., 2021). The topologies of the phylogenetic trees were evaluated with bootstrap analysis of 1,000 replicates (Felsenstein, 1985).

Whole genome sequencing was conducted on the Illumina HiSeq 4000 platform at Sangon Biotech (Shanghai, China). Sequence read quality was evaluated with FastQC (v.0.11.2) and then trimmed of adapters and low-quality sequence regions with Trimmomatic (v.0.36) (Bolger et al., 2014). The resultant sequence reads were assembled with SPAdes (v.3.5.0) (Bankevich et al., 2012). Gaps in assembled contigs were filled using GapFiller (v.1.11) (Boetzer and Pirovano, 2012) and corrected with PrinSeS-G (v.1.0.0) (Massouras et al., 2010). Genes were predicted using the Prokka annotation program (v.1.10) (Seemann, 2014). Repeat sequences were determined using RepeatModeler (v.2.0.2) and RepeatMasker (v.4.1.0). CRISPR prediction and analysis was conducted with CRT (v.1.2) (Bland et al., 2007). Genomic annotations were further analyzed by searches with the NCBI BLAST+ program (v.2.2.28) using default parameters. The digital DNA–DNA hybridization (dDDH) and average nucleotide identity (ANI) values between the genome of strain JXJ CY 39^T^ and those of other *Mucilaginibacter* type strains were calculated using the Genome-to-Genome Distance Calculator (GGDC version 3.0) (Meier-Kolthoff et al., 2013) and the JspeciesWS (JSWS) portal, respectively. The genomes of other type strains were downloaded from the NCBI genome database.^2^ DNA G + C% contents were calculated from genomic sequences. To construct a robust core gene phylogeny, a phylogenomic tree based on the concatenation of protein sequences from strain JXJ CY 39^T^ and the type strains of *Mucilaginibacter* was inferred with the EasyCGTree software package^3^ (Xue et al., 2021). Evolutionary distances were calculated with IQ-TREE (v.1.6.1) (Nguyen et al., 2015).

### 2.2 Co-culture of Maf and the attached bacteria

Eight other bacterial strains (Table 1) isolated from Maf cultures were used in the co-culture experiments in addition to strain JXJ CY 39^T^. Purified Maf (about 1.0 × 10^6^ CFU mL^−1^) was inoculated with attached bacteria in (i) binary culture systems (BCSs) that comprised Maf and an individual bacterium at a final cellular density of 1 × 10^6^ CFU mL^−1^ and (ii) ternary culture systems (TCSs) comprising Maf and strain JXJ CY 39^T^ in addition to another bacterium, with final cellular densities of both strains at 1 × 10^6^ CFU mL^−1^. Here, the purified Maf was cultured without supplement with additional bacteria, which served as the control. Both treatments (BCS and TCS) and controls included triplicate replicates. BCSs, TCSs, and controls were sampled at 5 and 10 days of cultivation. The bacterial cellular densities were determined using spread plate techniques based on the different features of the colors and morphologies of the colonies. Chlorophyll a (chl*-a*), extracellular microcystin LR (E-MC-LR), and intracellular microcystin LR (I-MC-LR) concentrations were measured using previously described methods (Zhang et al., 2015).

**Table 1:** The information of other 8 attached bacteria to Maf.

Strain no.	16S rRNA gene accession no.	The most related type strain	16S rRNA gene accession number of the type strain	Similarity (%)
JXJ CY 05	MZ708736	*Brevibacterium epidermidis* NBRC 14811^T^	BCSJ01000023	99.8
JXJ CY 11	OQ181347	*Pseudomonas oleovorans* ATCC 8062^T^	DQ842018	99.5
JXJ CY 16	OQ162229	*Agrococcus terreus* DNG5^T^	FJ423764	99.7
JXJ CY 18	MZ708737	*Methylorubrum thiocyanatum* DSM 11490^T^	AB175646	99.1
JXJ CY 28	MZ541062	*Sphingomonas abaci* C42^T^	AJ575817	99.6
JXJ CY 31	MZ708738	*Deinococcus wulumuqiensis* R12^T^	APCS01000185	99.9
JXJ CY 37	OQ162230	*Mycolicibacterium monacense* DSM 44395^T^	MVIA01000076	99.8
JXJ CY 57	MZ708739	*Pseudomonas toyotomiensis* DSM 26169^T^	AB453701	99.9

### 2.3 Nitrogen fixation and dissolution of unavailable phosphate

Nitrogen fixation ability was determined as previously described (Xiao et al., 2022b). The ability to dissolve insoluble phosphorus was evaluated using methods described by Zhang et al. (2016b). Both Ca_3_(PO_4_)_2_ (Damao Chemical Reagent Company, Tianjin, China) and phytin (Aladdin, Shanghai, China) were used as insoluble phosphorus sources at dosages of 1 g L^−1^. After growing on ISP2 agar plates at 28°C for 3 days, the cell mass of strain JXJ CY 39^T^ was collected and suspended using sterile tap-water, and the cellular suspension was served as the inoculum of nitrogen-free, Ca_3_(PO_4_)_2_ and phytin media.

### 2.4 Co-culture of Maf and JXJ CY 39^T^ in media with limited availability of N and P

Superfluous available nitrogen and phosphorus in eutrophic water are the critical element triggering cyanobacterial blooms. To assess nutrient provisioning activities, Maf at a density of 3 × 10^5^ CFU mL^−1^ and strain JXJ CY 39^T^ at a density of 1 × 10^6^ CFU mL^−1^ were co-cultured using modified BG11 media, where K_2_HPO_4_ (Damao Chemical Reagent Company, Tianjin, China) was replaced by Ca_3_(PO_4_)_2_ or NaNO_3_ (Damao Chemical Reagent Company, Tianjin, China) was removed to represent nitrogen-free media. Controls included purified Maf at a density of 3 × 10^5^ CFU mL^−1^ and strain JXJ CY 39^T^ at a density of about 1 × 10^6^ CFU mL^−1^ that were grown in modified BG11 media, respectively. Co-cultures and controls were all established in triplicate replicates. Bacterial cellular densities and the concentrations of chl-*a*, I-MC-LR, and E-MC-LR were examined as described above on days 7 and 14 for cultures in nitrogen-free medium and on days 9 and 18 for cultures in Ca_3_(PO_4_)_2_ medium. The inoculation of Maf into modified BG11 media was also added 1.86 mg L^−1^ of K_2_HPO_4_ into Ca_3_(PO_4_)_2_ medium and 71.4 mg L^−1^ of NaNO_3_ into nitrogen-free medium. The inoculum of strain JXJ CY 39^T^ was prepared as described above.

### 2.5 Influences of Maf metabolites on the growth of attached bacteria

To assess the influence of Maf metabolites on attached bacteria, Maf cultures (5 L, final cell density of ~2.5 × 10^7^ CFU mL^−1^) were distilled at 50°C under reduced pressure to remove water. The resultant condensates were extracted using mixture solvents comprising water: methanol: ethanol: ethyl acetate (1,1:1:1, *v*/*v*) and extracts were separated by medium pressure liquid chromatography with a C_18_ column (YMC, ODS-AQ, 50 μm) using stepwise elution of mixed solvents (methanol/water, 0/10 → 1/9 → 2/8 → 3/7 → 4/6 → 5/5 → 6/4 → 7/3 → 8/2 → 9/1 → 10/0, *v*/*v*). The resultant eluents were combined into four fractions based on HPLC detection results and were designated as fractions I, II, III, and IV, with quantities of 12.5, 1.75, 0.2, and 0.9 g, respectively. Fractions I, II, and III were water-soluble, while fraction IV was fat-soluble. Only fraction III contained MC-LR at a amount of about 6.2 mg, based on HPLC analyses. The inhibitory activities of these fractions on attached bacteria were investigated using the paper disk method, as previously described (Zhang et al., 2016a). The dosages of fractions I, II, and IV were 4 mg per disk, while disks with fraction III contained 4 μg MC-LR. Total Maf extracts were dissolved with deionized water and solutions were evaluated with antibacterial activities at 4 mg per disk.

### 2.6 Influence of strain JXJ CY 39^T^ on the growth of non-culturable Maf-attached bacteria

To assess the influences of strain JXJ CY 39^T^ and Maf on other Maf-attached bacteria, Maf and strain JXJ CY 39^T^ at a density of 1 × 10^6^ CFU mL^−1^ were co-cultured in BG11 medium as described above, followed by sampling at days 5, 10, 15 and 35 of cultivation (corresponding to sample IDs M39_1, M39_2, M39_3, and M39_4, respectively). Purified Maf cultures without strain JXJ CY 39^T^ were sampled at days 5, 10, 15, and 35 of cultivation and were considered the controls (C_1, C_2, C_3, and C_4). All of these samples were spread onto ISP2 plates and incubated at 28.0°C for 7 days to examine whether they were axenic. The V3–V4 hypervariable regions of 16S rRNA genes were amplified using the universal primers 338F (5′-ACTCCTACGGGAGGCAGCA-3′) and 806R (5′-GGACTACHVGGGTWTCTAAT-3′). The amplicons were sequenced on the Illumina MiSeq platform (Illumina, San Diego, USA) using standard protocols. Sequences were analyzed using the QIIME2 software package (2019.4) and by comparison of sequences to the Greengenes database (DeSantis et al., 2006). In addition, samples were inoculated onto ISP2 plates and cultured at 28.0°C for 5 to 7 days to assess the presence of viable strain JXJ CY 39^T^.

### 2.7 Statistics

Data were expressed as means ± standard deviations (*n* = 3). Comparisons of cellular densities, and concentrations of chl-*a* and MC-LR between co-cultures of Maf-bacteria and pure cultures of Maf or bacteria were performed using analysis of variance (ANOVA) followed by Tukey’s pairwise comparisons. Significance was set at *p* values of 0.05.

## 3 Results and discussion

### 3.1 Taxonomic study of strain JXJ CY 39^T^

#### 3.1.1 Morphological and physiological characteristics

Strain JXJ CY 39^T^ was aerobic, Gram-negative, and rod-shaped (0.5–0.8 × 0.9–2.0 μm) (Supplementary Figure S1). Its colonies were pale pink, smooth, convex, circular, and wet in appearance after 4–5 days of cultivation on IPS 2 agar plates. Growth occurred at 5.0–38.0°C, pH 4.0–11.0, and 0–3.0% NaCl, with optimal growth at 28.0°C, pH 7.0–8.0, and 0% NaCl. Strain JXJ CY 39^T^ was positive for catalase, oxidase, nitrate reduction, hydrolysis of starch, and Tween 40 and 80 tests, but negative for Tween 20. Additional phenotypic characteristics are shown in Table 2, along with the species description.

**Table 2:** Differential characteristics of strain JXJ CY 39^T^ and the closest type strains.

Characteristic	1	2	3
Isolation source	*M. aeruginosa*	Flower	Freshwater
Colony color	Pale pink	Pale orange	Pale pink
Cell size (μm)	0.5–0.7 × 1.0–2.0	0.7–0.8 × 1.5–2.5	0.5–0.7 × 1.1–1.8
Growth at (°C)	5–38 (28)	10–28	10–37 (30)
pH range for growth	4–11	6–8	6–8
Tolerance of NaCl (%, w/v)	0–3	0	0–2
Nitrate reduction	+	−	−
Starch hydrolysis	+	−	−
Assimilation of:			
_D_-Glucose	(+)	−	+
N-Acetyl-glucosamine	−	−	+
_D_-Mannose	(+)	−	+
_L_-Arabinose	−	−	+
Maltose	−	−	+
Acid production:			
_L_-Arabinose	−	−	+
_D_-Xylose	−	−	+
_D_-Galactose	−	−	+
_D_-Glucose	(+)	−	+
_D_-Fructose	−	−	+
_D_-mannose	(+)	−	+
_L_-Rhamnose	−	−	+
_D_-Cellobiose	−	−	+
_D_-Maltose	(+)	−	+
_D_-Melibiose	−	−	+
_D_-Sucrose	−	−	+
_D_-Trehalose	−	−	+
_D_-Raffinose	−	−	+
Enzyme activity (API ZYM):			
Cystine arylamidase	+	+	−
Trypsin	+	−	−
α-Mannosidase	+	−	−
α-Fucosidase	+	−	−
Genomic characteristics			
CheckM completeness	98.1%	98.1%	98.1%
CheckM contamination	1.9%	1.9%	1.19%
CheckM strain heterogeneity	0.0	0.0	0.0
5S count	1	3	1
16s count	1	3	1
23S count	1	3	1
tRNA count	18	19	19
Contig count	24	2	10
N50 contigs	1,543,143 bp	4,896,009 bp	729,309 bp
Longest contig	1,553,743 bp	4,896,009 bp	988,577 bp
Scaffold count	24	2	10
N50 scaffolds	1,543,143 bp	4,896,009 bp	729,309 bp
Longest scaffold	1,553,743 bp	4,896,009 bp	988,577 bp
Genome size	5,064,077 bp	4,984,039 bp	4,330,477 bp
Protein count	4,424	4,370	3,759
Coding density	89.24%	88.57%	90.70%
GC percentage	42.09%	41.39%	41.63%
Ambiguous bases	11	0	0
GTDB representative	GCA_009770985.1	GCA_012849215.1	GCA_009755275.1

1, JXJ CY 39^T^; 2, *M. robiniae* F39- 2^T^ (Won et al., 2022); 3, *M. aquatilis* HME9299^T^ (Kang et al., 2021). +, positive; −, negative; (+), weak positive. All of these strains are rod-shaped; positive for catalase, oxidase, acid and alkaline phosphatase, lipase C4,8, α-Galactosidase and β-Glucosidase; negative for α-Chymotrypsin and β-Glucuronidase.

#### 3.1.2 Chemotaxonomy

The major cellular fatty acids of strain JXJ CY 39^T^ were iso-C_15:0_ (45.0%) and C_16:1_ω7c/_16:1_ω6c (30.3%) (Supplementary Table S1) and the predominant menaquinone was MK-7, while the polar lipids comprised phosphatidylethanolamine (PE), unidentified aminophosphoglycolipid (APGL), unidentified aminoglycolipids (AGL), unidentified phospholipid (PL), and unidentified polar lipids (L1-3) (Supplementary Figure S2). The above characteristics were similar to the chemotaxonomic profile of *M. robiniae* F39- 2^T^ (Won et al., 2022) and *M. aquatilis* HME9299^T^ (Kang et al., 2021).

#### 3.1.3 Molecular phylogenetic analysis

A nearly complete 16S rRNA gene sequence (1,509 bp length; GenBank accession MT674523) of strain JXJ CY 39^T^ exhibited close similarities with *Mucilaginibacter robiniae* F39-2^T^ (97.02%), *Mucilaginibacter aquatilis* HME9299^T^ (96.69%), *M. galii* PP-F2F-G47^T^ (96.50%), *M. straminoryzae* RS28^T^ (96.10%), and *M. polytrichastri* DSM 26907^T^ (96.05%), respectively, and < 96% similarities with other *Mucilaginibacter* spp. and these strains formed a distinct clade within the *Mucilaginibacter* lineage in the phylogenetic trees of 16S rRNA gene sequences (Supplementary Figures S3–S5), which was recapitulated in the Maximum-Likelihood core gene phylogenomic tree (Supplementary Figure S6). The dDDH and ANI values between strain JXJ CY 39^T^ and its phylogenetic neighbors were 18.8–19.9% and 70.43–73.95%, respectively. These values are all much lower than the generally accepted species threshold values (Chun et al., 2018). Therefore, based on the phenotypic, genotypic and phylogenetic properties, strain JXJ CY 39^T^ represents a novel species of the genus *Mucilaginibacter*, for which the name *Mucilaginibacter lacusdianchii* sp. nov. is proposed.

### 3.2 Genomic characteristics

The genome of strain JXJ CY 39^T^ was sequenced and submitted to GenBank under the accession ID WSRW00000000. The genome of strain JXJ CY39^T^ contains six contigs, with a total length of 5,070,224 bp and an N_50_ length of 1,554,798 bp. The genomic characteristics of the closest reference strains *M. robiniae* F39- 2^T^ (Won et al., 2022) and *M. aquatilis* HME9299^T^ (Kang et al., 2021) were included in Table 2.

In the genome of strain JXJ CY 39^T^, some genes or gene clusters have been identified that are likely to enhance symbiotic interactions with the Maf (Supplementary Tables S2, S3). GO database annotation of genes indicated that strain JXJ CY 39^T^ encoded 19 gene clusters related to symbiotic interactions, immune responses, and their regulation (Supplementary Table S2); 3 gene clusters related to plant growth hormone synthesis (e.g., of indole-3-acetic acid, auxin, and polyamine); 13 gene clusters related to the synthesis of various vitamins (e.g., B_1_, B_2_, B_6_, B_12_, K_2_, and biotin) and their derivatives; 4 genes related to ATP-binding cassette (ABC) transporter complexes; 8 genes related to protein secretion; over 26 gene clusters encoding various glycosidases; 76 genes related to carbohydrate catabolism; 44 genes related to phosphatases activity; 185 genes related to organic acid biosynthesis; 22 genes related to organic acid transport; 11 genes related to nodulation; 6 genes related to nitrogen fixation; 3 genes related to catalase activity; and 14 genes related to peroxidases; 7 genes related to carotenoid biosynthetic process (Supplementary Table S3).

The interconnected evolutionary histories of Cyanobacteria and bacteria have led to the formation of various interactions between the two, including through nutrient exchange, signal transduction, gene transfer, and inhibition (Kouzuma and Watanabe, 2015; Cirri and Pohnert, 2019). For example, protein secretion systems and ATP-binding cassette (ABC) transporters are involved in the exchange of substances and signal transduction between cyanobacteria and their attached bacteria (Zhu et al., 2021). Consequently, the above genomic data for strain JXJ CY 39^T^ suggest the potential for exchange of substances and signal transduction between the bacteria and Maf, prompting follow-on experiments to evaluate the presence of such interactions.

### 3.3 Co-culture growth of Maf and strain JXJ CY 39^T^

**Figure 1:**
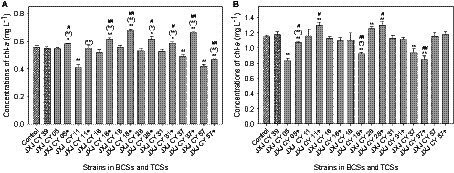
Influence of BCSs and TCSs on Maf growth. **(A,B)** Represent samples collected at 5 and 10 days of cultivation, respectively. +, indicates addition of strain JXJ CY 39^T^ in the culture. Error bars indicate standard deviation based on measurements of three replicates. * and ** indicate statistically significant differences in measurements between control cultures and BCS (or TCS) at *p* < 0.05 and *p* < 0.01, respectively. (*) and (**) indicate statistically significant differences in measurements between relevant BCSs and TCSs at *p* < 0.05 and *p* < 0.01, respectively. # and ## indicate statistically significant differences between measurements of BCS with JXJ CY 39^T^ cultures and relevant TCSs at *p* < 0.05 and *p* < 0.01, respectively.

The chl-*a* concentrations of the control increased from 0.092 mg L^−1^ to 0.555 and 1.149 mg L^−1^ after 5 and 10 days of cultivation, respectively. Six of the attached bacteria in the BCSs did not influence the growth of Maf after 5 days of cultivation (*p* > 0.05). The exceptions were strains JXJ CY 11, 37, and 57 that resulted in decreased chl-*a* concentrations of 26.1, 11.6, and 24.6% (*p* < 0.01), respectively (Figure 1A). After 5 days of cultivation, the chl-*a* concentration of TCS with strains JXJ CY 57 + 39 was 0.466 mg L^−1^ or 16.1% lower (*p* < 0.01) than that of the control. The chl-*a* concentration of TCS with strains JXJ CY 11 + 39 was not different from that of the control (*p* > 0.05), while the chl-*a* concentrations of the other six TCSs were 0.580–0.678 mg L^−1^ or 4.5–22.1% higher (*p* < 0.05, *p* < 0.01) than that of the control (Figure 1A). Moreover, all chl-*a* concentrations of TCSs were 6.7–34.7% higher (*p* < 0.05, *p* < 0.01) than those of related BCSs after 5 days of cultivation, while six of the chl-*a* concentrations of TCSs were 5.6–23.4% higher (*p* < 0.05, *p* < 0.01) than that of BCS with JXJ CY 39^T^ (Figure 1A).

After 10 days of culture, the chl-*a* concentrations of BCSs with strain JXJ CY 05 and 37 were 0.834, and 0.940 mg L^−1^, representing 27.4 and 18.2% lower (*p* < 0.01) than that of the control, respectively (Figure 1B). However, the chl-*a* concentration of BCS with JXJ CY 28 was 1.26 mg L^−1^ or 9.2% higher (*p* < 0.01) than that of the control (Figure 1B). In addition, the chl-*a* concentrations of TCSs with strains JXJ 05 + 39, 18 + 39, and 37 + 39 were 1.069, 0.921, and 0.848 mg L^−1^ after 10 days of cultivation, representing 7.0, 19.9, and 26.2% lower (*p* < 0.01) than that of the control. While, the chl-*a* concentrations of TCSs with strains JXJ CY 11 + 39 and 28 + 39 were 1.292 and 1.300 mg L^−1^ after 10 days of cultivation, representing 12.4 and 13.1% higher (*p* < 0.01) than that of the control, respectively. The chl-*a* concentrations of TCSs with strains JXJ CY 05 + 39 and 18 + 39 were 28.1% higher (*p* < 0.01) and 16.7% lower (*p* < 0.05) than those of the relevant BCSs, respectively.

The chl-*a* concentrations of TCSs with strains JXJ CY 05 + 39, 18 + 39, and 37 + 39 were 9.0, 21.6, and 27.8% lower (*p* < 0.05, *p* < 0.01) than that of the BCS with strain JXJ CY 39^T^, respectively; while the chl-*a* concentrations of TCSs with strains JXJ CY 11 + 39 and 28 + 39 were 10.0 and 10.7% higher (*p* < 0.05) than that of BCS with JXJ CY 39^T^, respectively. These data indicate that various strains may have distinct effects on Maf growth and that all adherent bacteria probably affect the interactions between Maf and other adherent bacteria, corroborating our previous research (Xiao et al., 2022c).

Algal–bacterial mutualisms are largely mediated by nutrition provisioning from bacteria (Cooper and Smith, 2015). Further, algal growth can be promoted by plant hormones, like indole-3-acetic acid (IAA) (Cirri and Pohnert, 2019; Hoke et al., 2021) and auxin (Seyedsayamdost et al., 2011). In addition, *Microcystis* requires vitamin B_12_ for methionine biosynthesis and many other vitamins for growth including B_1_ and B_7_ (Hoke et al., 2021). Gene annotations (Supplementary Table S3) indicated that strain JXJ CY 39^T^ could potentially provide Maf with plant growth hormones and various vitamins. Such mutualistic behaviors are likely the reason that the chl-*a* concentrations of some co-cultures were higher than those of the controls grown in BG11 medium (Figure 1). Kim et al. (2019) also reported that attached bacteria can promote Maf growth by decomposing H_2_O_2_ under aerobic growth conditions. Strain JXJ CY 39^T^ encodes catalases (Table 2; Supplementary Table S3) and peroxidases (Supplementary Table S3). Consequently, the decomposition of H_2_O_2_ produced by Maf could be another mechanism by which strain JXJ CY 39^T^ promotes the growth of Maf. Attached bacteria could provide *Microcystis* with complementary carotenoid molecules (Pérez-Carrascal et al., 2021). Carotenoids play an important role in protecting chlorophyll molecules against photo-oxidative damage (Young, 1991). Strain JXJ CY 39^T^ has 7 genes related to carotenoid biosynthetic process, indicating that this strain can also potentially protect chlorophyll molecules of Maf against photo-oxidative damage and promote the photosynthetic efficiency of the algae, which is probably another reason of JXJ CY 39^T^ potentially promoting the growth of Maf.

Interactions between algae and attached bacteria are dynamic and can be initiated and ended in response to environmental and developmental cues (Cooper and Smith, 2015). Such dynamism was apparent from the influences of attached bacteria on Maf growth. For example, the influences of attached bacteria on Maf growth in BCSs with strains JXJ 05, 11, 28, and 57, and in TCSs with strains 05 + 39, 11 + 39, 16 + 39, 18 + 39, 31 + 39, 37 + 39, and 57 + 39 changed with cultivation time (Figure 1). Almost the similar phenomena were also observed in previous studies (Zhang et al., 2016b,c, 2017; Xiao et al., 2022a,b,c).

Cellular densities of attached bacteria increased significantly (*p* < 0.05) after 5 days of cultivation (Table 3), except for those of strains JXJ CY 16, 18, 28, and 31 in BCSs, and strain JXJ CY 39^T^ in TCSs with JXJ CY 05 + 39 and 37 + 39, and strains JXJ CY 28 and 31 in TCSs. The cellular densities then significantly decreased (*p* < 0.01) with culture time. Further, strain JXJ CY 31 was even not detected on day 10 of BCS cultivation nor on days 5 and 10 of cultivation in TCS, while strain JXJ CY 39^T^ was not detected in both BCS and TCSs on the day 10 of cultivation. The cellular densities of the other eight bacteria in TCSs were significantly influenced (*p* < 0.01) by cultivation with strain JXJ CY 39^T^ on both days 5 and 10 of cultivation. However, the cellular densities of JXJ CY 39^T^ in TCSs were only influenced by cultivation with strains JXJ CY 05, 16, 31, 37, and 57 on day 5 of cultivation (*p* < 0.005, *p* < 0.001), but were not influenced by cultivation with any of the other eight strains on day 10 of cultivation (*p* > 0.05). Thus, the influences of Maf on attached bacteria likely varies with bacterial strain and cultivation duration. Moreover, all attached bacteria would likely influence the interactions between Maf and other bacterial taxa. These results are consistent with those from our previous study (Xiao et al., 2022c), and also reflect the dynamic interactions between algae and attached bacteria.

**Table 3:** Cell densities of nine strains in BCS and TCS.

Strains	Cell densities (CFU mL^−1^)					
	BCS		TCS		JXJ CY 39 in TCS	
	Day 5	Day 10	Day 5	Day 10	Day 5	Day 10
JXJ CY 39	1.38 ± 0.17 × 10^6*^	0				
JXJ CY 05	1.01 ± 0.11 × 10^7**^	8.33 ± 0.85 × 10^5**^	9.03 ± 0.51 × 10^6**##^	1.11 ± 0.15 × 10^5**##^	1.02 ± 0.13 × 10^6#^	0^**^
JXJ CY 11	1.82 ± 0.21 × 10^7**^	3.77 ± 0.49 × 10^6**^	4.62 ± 0.38 × 10^7**##^	2.19 ± 0.32 × 10^7**##^	1.19 ± 0.12 × 10^6*^	0^**^
JXJ CY 16	5.27 ± 0.76 × 10^5**^	7.33 ± 1.53 × 10^2**^	3.57 ± 0.45 × 10^6**##^	1.39 ± 0.14 × 10^4**##^	2.37 ± 0.61 × 10^6*^	0^**^
JXJ CY 18	1.11 ± 0.10 × 10^6^	1.59 ± 0.18 × 10^4**^	2.01 ± 0.22 × 10^6**##^	2.73 ± 0.32 × 10^4**##^	1.43 ± 0.15 × 10^6**^	0^**^
JXJ CY 28	5.57 ± 0.72 × 10^4**^	1.82 ± 0.14 × 10^4**^	1.82 ± 0.17 × 10^5**##^	4.83 ± 0.35 × 10^4**##^	1.62 ± 0.11 × 10^6**^	0^**^
JXJ CY 31	1.02 ± 0.13 × 10^5**^	0^**^	0^**##^	0	2.83 ± 0.26 × 10^6**##^	0^**^
JXJ CY 37	3.73 ± 0.61 × 10^7**^	1.11 ± 0.18 × 10^6**^	4.15 ± 0.33 × 10^6**##^	3.37 ± 0.15 × 10^5**##^	8.77 ± 0.86 × 10^5#^	0^**^
JXJ CY 57	3.03 ± 0.20 × 10^7**^	1.39 ± 0.15 × 10^7**^	3.86 ± 0.17 × 10^7**##^	1.83 ± 0.16 × 10^6**##^	1.85 ± 0.18 × 10^6**#^	0^**^

* and ** indicate statistically significant differences of measurements between days 0 and 5 or days 5 and 10 of cultivation in BCSs (or TCSs) at *p* < 0.05 and *p* < 0.01, respectively. # and ## indicate statistically significant differences between measurements from relevant BCSs and TCSs at *p* < 0.05 and *p* < 0.01, respectively.

BG11 medium is a synthetic medium that lacks sufficient organic carbon sources, such that heterotrophic attached bacteria will not grow due to insufficient carbon and energy sources. Dissolved organic carbon secreted by *M. aeruginosa* (Casamatta and Wickstrom, 2000) can be used by bacteria for growth. A previous study showed that bacteria attached to *Microcystis* cells encode higher relative abundances of carbon degradation genes and β-glucosidase activity to enable the use of organic carbon secreted by *Microcystis* (Yang et al., 2021). Annotation of the strain JXJ CY 39^T^ genome revealed the presence of many genes that would enable use of dissolved organic carbon secreted by Maf (Supplementary Table S3). This likely explains why the attached bacteria grew during the first five days despite the organic carbon-deficient medium used for cultivation.

The analysis of metagenomes, metatranscriptomes and metaproteomes on natural water bloom samples play important roles in the recognition of interactions in phytoplankton communities (Kazamia et al., 2016). However, these technologies can only let us know the overall metabolic capability and ecological state of a community (Kazamia et al., 2016), and many aspects of these interactions, especially specific interactions of the microbes within, are still unknown (Grossart and Simon, 2007; Kazamia et al., 2016; Zhang et al., 2019) because of most of these studies were performed under non-axenic conditions (Grossart and Simon, 2007; Zhang et al., 2019). Specific interactions can only probably be understood through physiological experiments using defined systems such as co-cultures of known and well-characterized partners in the laboratory, which can shed light on the interactions at the molecular and cellular levels (Kazamia et al., 2016). In this study, we have removed other culturable heterotrophic bacteria from Maf, and then inoculated the specific pure cultures of these isolated attached bacteria back to Maf, which can eliminate the distractions of other unknown culturable heterotrophic bacteria on the specific interactions, and let us know more about the interactions between Maf and its attached bacteria like mentioned above and below.

### 3.4 Influences of co-cultures on MC-LR concentrations

**Figure 2:**
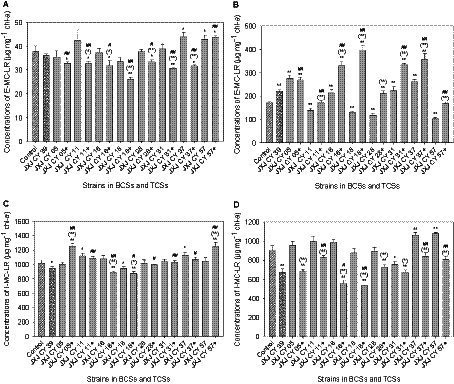
Influence of BCSs and TCSs on E-MC-LR and I-MC-LR concentrations in Maf cultures. **(A,C)** Represent samples collected at 5 day of cultivation. **(B,D)** Represent samples collected at 10 day of cultivation. + indicates strain JXJ CY 39^T^ was included in the co-culture. Error bars indicate standard deviations for measurements from three replicates. * and ** indicate statistically significant differences of measurements between control cultures and BCSs (or TCSs) at *p* < 0.05 and *p* < 0.01, respectively. (*) and (**) indicate statistically significant differences between measurements from relevant BCSs and TCSs at *p* < 0.05 and *p* < 0.01, respectively. # and ## indicate statistically significant differences between measurements of BCS with JXJ CY 39^T^ and relevant TCSs at *p* < 0.05 and *p* < 0.01, respectively.

The E-MC-LR concentrations of the control were 37.6 and 172.2 μg mg^−1^ chl-*a* on days 5 and 10 of cultivation, respectively. The E-MC-LR concentrations of BCSs with strains JXJ CY 37 and 57, in addition to TCS with strains JXJ CY 57 + 39 on day 5 of cultivation were 44.0, 42.9, and 43.5 μg mg^−1^ chl-*a*, respectively, representing 17.0, 14.1 and 15.6% higher (*p* < 0.05) values compared to controls, respectively. However, the E-MC-LR concentrations of the other seven BCSs did not significantly differ from the control, while the E-MC-LR concentrations of the other seven TCSs were 11.3–30.5% lower (*p* < 0.05, *p* < 0.01) than that of the control (Figure 2A). Moreover, the E-MC-LR concentrations of the six TCSs were 11.4–28.2% lower (*p* < 0.05, *p* < 0.01) than those of the relevant BCSs (Figure 2A). The E-MC-LR concentrations of the TCSs were 7.6–27.6% lower (*p* < 0.05, *p* < 0.01) than that of BCS with strain JXJ CY 39, with the exception of TCS with strain JXJ CY 57 + 39 that was 20.4% higher (*p* < 0.01) (Figure 2A).

After day 10 of cultivation, the E-MC-LR concentrations of BCSs with strains JXJ CY 11, 18, 28, and 57 were 19.7, 23.6, 31.8, and 40.5% lower (*p* < 0.01) than that of the control, respectively; while the E-MC-LR concentrations of BCSs with strains JXJ CY 05, 16, 31, 37, and 39 were 60.8, 24.8, 29.7, 51.3, and 27.9% higher (*p* < 0.01) than that of the control, respectively (Figure 2B). At 10 days of cultivation, the E-MC-LR concentrations of the six TCSs were 21.7–129.8% higher (*p* < 0.05, *p* < 0.01) than the control, with the exception of TCSs containing strains JXJ CY 11 + 39 and 57 + 39 (Figure 2B). The E-MC-LR concentrations of the seven TCSs were 24.4–200.9% higher (*p* < 0.01) than those of relevant BCSs, with the exception of the TCS with strains JXJ CY 05 + 39 (Figure 2B). The E-MC-LR concentrations of TCSs with strains JXJ CY 11 + 39 and 57 + 39 were 21.9–24.3% lower (*p* < 0.01) than that of BCS with strain JXJ CY 39, while the E-MC-LR concentrations of the other TCSs were 22.2–79.7% higher (*p* < 0.05) than that of BCS with strain JXJ CY 39, with the exception of the TCS with strain JXJ CY 28 + 39 (Figure 2B). Thus, the different strains exerted different and dynamic influences on the E-MC-LR concentrations of Maf. Moreover, the results suggest that the addition of any strain into a BCS with one attached bacterium would likely influence the E-MC-LR concentrations of Maf. These results are consistent with previous study (Xiao et al., 2022c).

The I-MC-LR concentrations of the controls were 1,019.0 and 900.8 μg mg^−1^ chl-*a* on days 5 and 10 of cultivation, respectively (Figures 2C, D). On day 5 of cultivation, the I-MC-LR concentrations of BCSs with strains JXJ CY 11 and 37 were 1,117.8, and 1,130.7 μg mg^−1^ chl-*a*, representing 9.7 and 11.0% higher (*p* < 0.05) than that of the control, respectively. In addition, the I-MC-LR concentrations of BCSs with strains JXJ CY 18 and 39 were 946.9 and 950.1 μg mg^−1^ chl-*a*, respectively, representing 7.1 and 6.8% lower (*p* < 0.05) than that of the control, respectively (Figure 2C). After 5 days of culture, the I-MC-LR concentrations of TCSs with strains JXJ CY 05 + 39 and 57 + 39 were 25.4 and 19.1% higher (*p* < 0.01) than those of relevant BCSs, respectively. In addition, the I-MC-LR concentrations of TCSs with strains JXJ CY 16 + 39 and 18 + 39 were 17.2 and 7.2% lower (*p* < 0.05, *p* < 0.01) than those of the related BCSs, respectively. The I-MC-LR concentrations of TCSs were 4.6–32.1% higher (*p* < 0.05, *p* < 0.01) than that of BCS with JXJ CY 39, with the exception of TCSs with strains JXJ CY 16 + 39 and 18 + 39 that exhibited 5.8 and 7.6% lower (*p* < 0.01, *p* < 0.05) than that of BCS with strain JXJ CY 39, respectively (Figure 2C).

On day 10 of cultivation, the I-MC-LR concentrations of BCSs with strains JXJ CY 39 and 31 were 25.2 and 16.0% lower (*p* < 0.01, *p* < 0.05) than the control, respectively, and the I-MC-LR concentrations of BCSs with strains JXJ CY 37 and 57 were 18.0 and 19.6% higher (*p* < 0.01) than the control (Figure 2D), respectively. On day 10 of cultivation, the I-MC-LR concentrations of the TCSs were 11.4–43.5% lower (*p* < 0.05, *p* < 0.01) than those of the related BCSs, and 19.1–40.6% lower (*p* < 0.01) than the control, with the exception of TCSs with strains JXJ CY 11 + 39, 37 + 39, and 57 + 39 that were not different from the control (*p* > 0.05) (Figure 2D). The I-MC-LR concentrations of TCSs with strains JXJ CY 16 + 39 and 18 + 39, in addition to TCSs with strains JXJ CY 11 + 39, 37 + 39, and 57 + 39 were 17.5–20.6% lower (*p* < 0.05, *p* < 0.01) and 19.9–24.9% higher (*p* < 0.01) than that of the BCS with strain JXJ CY 39 (Figure 2D), respectively. While the I-MC-LR concentrations of the TCSs with JXJ 05 + 39 on day10 of cultivation was 24.2% lower (*p* > 0.01) than the control (Figure 2D) in contrast to that of TCSs with JXJ 05 + 39 on day 5 of cultivation, which was 23.2% higher (*p* > 0.01) than the control (Figure 2C). These data indicate that different strains probably exert different and dynamic influences on the I-MC-LR concentrations of Maf. Moreover, the addition of any strain into a BCS with one attached bacterium would also likely influence the I-MC-LR concentrations of Maf. These results are consistent with our previous study (Xiao et al., 2022c).

### 3.5 Capacity for nitrogen fixation and phosphate acquisition

Cellular densities of strain JXJ CY 39^T^ initially increased from 1 × 10^5^ CFU mL^−1^ to 3.9 × 10^6^ CFU mL^−1^ after 2 days of cultivation in nitrogen-free medium, indicating that this strain could convert N_2_ into NH_3_ to support its growth, which was consistent with annotation of the strain JXJ CY 39^T^ genome with the GO database (Supplementary Table S3). After 2 days of co-cultivation, the concentrations of available phosphorus increased by 10.41 ± 0.44 mg L^−1^ for Ca_3_(PO_4_)_2_ and 5.68 ± 0.24 mg L^−1^ for phytin, indicating that strain JXJ CY 39^T^ can dissolve insoluble inorganic and organic phosphate, and release dissoluble phosphorus, consistent with the genomic analyses (Supplementary Table S3). Therefore, strain JXJ CY 39^T^ can potentially provide Maf with available N and P when available N and P are limited in the environment.

### 3.6 Influences of limited P and N availability on Maf and JXJ CY 39^T^ growth

In nitrogen-deficient medium, the color of Maf cultured without JXJ CY39^T^ turned green gradually during the first 7 days, and turned yellow quickly during the second 7 days. Consequently, chl-*a* concentrations of Maf cultured without JXJ CY39^T^ increased to 0.371 mg L^−1^ on day 7 from 0.030 mg L^−1^ on day 0, and then decreased to 0.176 mg L^−1^ on day 14 (Table 4). However, in nitrogen-deficient medium, the color of Maf co-cultured with JXJ CY39^T^ turned green more quickly during the first 7 days, and turned yellow more slowly during the second 7 days. Consequently, chl-*a* concentrations of Maf co-cultured with JXJ CY39^T^ increased to 0.564 mg L^−1^ on day 7, which was 52.0% higher (*p* < 0.01) than that of the control, and then decreased to 0.463 mg L^−1^ on day 14, which was 163.1% higher (*p* < 0.01) than that of the control (Table 4).

**Table 4:** Influences of limited available N on the growths of Maf and JXJ CY 39^T^.

Groups	Targets	On day 7	On day 14
a	Chl-*a* (mg L^−1^)	0.371 ± 0.013	0.176 ± 0.008^(**)^
	E-MC-LR (μg mg^−1^ chl-*a*)	98.4 ± 3.8	339.4 ± 23.9^(**)^
	I-MC-LR (μg mg^−1^ chl-*a*)	968.2 ± 42.6	1162.2 ± 39.2^(**)^
b	Chl-*a* (mg L^−1^)	0.564 ± 0.026^**^	0.463 ± 0.013^**(**)^
	E-MC-LR (μg mg^−1^ chl-*a*)	45.3 ± 0.9^**^	159.8 ± 5.7^**(**)^
	I-MC-LR (μg mg^−1^ chl-*a*)	942.1 ± 27.5	965.5 ± 31.8^**^
	Cell densities of JXJ CY 39^T^ (CFU mL^−1^)	2.93 ± 0.31 × 10^6##^	3.27 ± 0.47 × 10^6##^
c	Cell densities of JXJ CY 39^T^ (CFU mL^−1^)	9.07 ± 0.50 × 10^4^	2.03 ± 0.25 × 10^4(**)^

a, Maf cultured with no strain JXJ CY 39^T^; b, Maf cocultured with strain JXJ CY 39^T^; c, strain JXJ CY 39^T^ cultured with no Maf. *, ** indicate the significant differences between a and b at the level of *p* < 0.05, *p* < 0.01, respectively, at the same culture time. (**) indicated the significant differences between day 7 and day 14. ## indicate the significant differences between b and c at the level of *p* < 0.05, *p* < 0.01, respectively, at the same culture time.

As mentioned above, the inoculation of Maf cultured in BG11 imported 71.4 mg L^−1^ of NaNO_3_ into nitrogen-free medium, in addition to 0.321 mg L^−1^ of N as NH_4_^+^ in the ferric ammonium citrate. Additionally, degradation of phycobiliproteins of *Microcystis* can expand biomass by about 50% (Wang et al., 2021). These available nitrogen sources were the reasons of the growth of Maf cultured without JXJ CY39^T^ during the first 7 days in nitrogen-free medium. However, exhausting these available nitrogen sources resulted in mass mortality of Maf, which was the main reason that the color of Maf cultured without JXJ CY39^T^ turned yellow quickly during the second 7 days.

Ammonia is a key component involved in aquatic environment microbial interactions (Cirri and Pohnert, 2019), and can dissolve in water easily and form NH_3_·H_2_O, which further ionizes and produces NH_4_^+^. *Microcystis* prefer ammonium (NH_4_^+^) because of its attached bacteria often lacking functional genes that mediate nitrification (Yang et al., 2021). Therefore, strain JXJ CY39^T^ can provide Maf with NH_4_^+^ since it can convert N_2_ into NH_3_, which was the main reason that Maf co-cultured with JXJ CY39^T^ expand more biomass during the first 7 days, and exhaust lesser biomass during the second 7 days (Table 4). Therefore, strain JXJ CY 39^T^ can provide Maf with available N when available N is limited. It is consistent with those of other co-cultures with other attached bacteria and Maf (Xiao et al., 2022a,b,c).

In nitrogen-deficient medium, strain JXJ CY39^T^ also influenced the synthesis of MC-LR (Table 4). The E-MC-LR concentrations of Maf co-cultured with JXJ CY39^T^ were 45.3 and 159.8 μg mg^−1^ chl-*a*, which were 54.0, and 52.9% lower (*p* < 0.01) than those of the controls. The I-MC-LR concentration of Maf co-cultured with strain JXJ CY39^T^ was 1,162.2 μg mg^−1^ chl-*a* on day 14 of cultivation, which was 16.9% lower (*p* < 0.01) than that of the control. Similar phenomena were also found in previous studies (Xiao et al., 2022a,b).

In Ca_3_(PO_4_)_2_ medium, the color of Maf cultured without strain JXJ CY39^T^ turned green gradually during the test time. Consequently, the chl-*a* concentrations increased to 0.639, and 0.985 mg L^−1^ after 9 and 18 days of culturation (Table 5), respectively. However, the color of Maf co-cultured with strain JXJ CY39^T^ turned greener during the test time, and the chl-*a* concentrations increased to 0.745, and 1.163 mg L^−1^ after 9 and 18 days of culturation, which were 16.6, and 18.1% higher (*p* < 0.01) than those of the controls (Table 5), respectively.

**Table 5:** Influences of limited available P on the growths of Maf and JXJ CY 39^T^.

Groups	Targets	On day 9	On day 18
a	Chl-*a* (mg L^−1^)	0.639 ± 0.032	0.985 ± 0.017^(**)^
	E-MC-LR (μg mg^−1^ chl-*a*)	16.0 ± 0.9	56.1 ± 2.5^(**)^
	I-MC-LR (μg mg^−1^ chl-*a*)	1133.0 ± 26.9	1949.6 ± 17.6^(**)^
b	Chl-*a* (mg L^−1^)	0.745 ± 0.008^**^	1.163 ± 0.022^**(**)^
	E-MC-LR (μg mg^−1^ chl-*a*)	19.6 ± 0.8^**^	47.9 ± 6.2^(**)^
	I-MC-LR (μg mg^−1^ chl-*a*)	1022.3 ± 40.6^*^	1713.1 ± 22.8^**(**)^
	Cell densities of JXJ CY 39^T^ (CFU mL^−1^)	7.27 ± 0.71 × 10^4##^	1.46 ± 0.11 × 10^5##(**)^
c	Cell densities of JXJ CY 39^T^ (CFU mL^−1^)	1.43 ± 0.25 × 10^4^	1.77 ± 0.42 × 10^3(**)^

a, Maf cultured with no strain JXJ CY 39^T^; b, Maf cocultured with strain JXJ CY 39^T^; c, strain JXJ CY 39^T^ cultured with no Maf. *, ** indicate the significant differences between a and b at the level of *p* < 0.05, *p* < 0.01, respectively, at the same culture time. (**) indicated the significant differences between day 9 and day 18. ## indicate the significant differences between b and c at the level of *p* < 0.05, *p* < 0.01, respectively, at the same culture time.

As mention above, the inoculation of Maf cultured in BG11 imported 1.86 mg L^−1^ of K_2_HPO_4_ into Ca_3_(PO_4_)_2_ medium. And Ca_3_(PO_4_)_2_ is slightly soluble in water and breaks down into Ca^2+^ and PO_4_^3+^. Therefore, dissoluble phosphorus is the key element that restricts the growth of Maf in Ca_3_(PO_4_)_2_ medium, in spite of the uptake of PO_4_^3+^ by Maf would result in more Ca_3_(PO_4_)_2_ being dissolved. These were the reasons that Maf cultured without strain JXJ CY39^T^ turned green more slowly. Secreting phosphatases and organic acids are two of the important mechanisms that microbes degrade insoluble phosphorus into dissoluble phosphorus. Therefore, strain JXJ CY39^T^ can facilitate the dissolving of Ca_3_(PO_4_)_2_ since it has abundant genes related to phosphatase activity, and organic acid biosynthetic process and transmembrane transport (Supplementary Table S3), which would relieve the restriction of lacking dissoluble phosphorus on the growth of Maf in Ca_3_(PO_4_)_2_ medium. This was the main reason that Maf co-cultured with strain JXJ CY39^T^ expanded more biomass than Maf cultured without strain JXJ CY39^T^ in Ca_3_(PO_4_)_2_ medium (Table 5). These results were consistent with previous studied of co-culture of other phosphate-solubilizing attached bacteria and *Microcystis* (Xiao et al., 2022a,b,c).

Strain JXJ CY39^T^ also significantly influenced MC-LR synthesis of Maf in Ca_3_(PO_4_)_2_ medium (Table 5). The E-MC-LR concentration of Maf co-cultured with strain JXJ CY39^T^ was 19.6 μg mg^−1^ chl-*a* on day 9 of cultivation, which was 22.5% higher (*p* < 0.01) than that of the control. The I-MC-LR concentrations of Maf cultured with strain JXJ CY39^T^ were 1,022.3 and 1,713.1 μg mg^−1^ chl-*a* on days 9 and 18 of cultivation, representing 9.8 and 12.1% lower (*p* < 0.05, *p* < 0.01) than those of the controls, respectively. Similar phenomena were also found in previous studies (Xiao et al., 2022a,b).

The cellular densities of strain JXJ CY39^T^ cultured without Maf decreased with the cultivation time (*p* < 0.01) in media with both Ca_3_(PO_4_)_2_ and nitrogen limitation (Tables 4, 5). However, the cellular densities of strain JXJ CY39^T^ co-cultured with Maf in available nitrogen-deficient medium increased to 2.93 × 10^6^ CFU mL^−1^ after 7 days of cultivation, and maintained about 3 × 10^6^ CFU mL^−1^during the next 7 days, which were over 32–161-fold higher (*p* < 0.01) than those cultured without Maf in nitrogen-deficient medium (Table 4). Further, the cellular densities of strain JXJ CY39^T^ co-cultured with Maf in Ca_3_(PO_4_)_2_ medium were 7.27 × 10^4^ and 1.46 × 10^5^ CFU mL^−1^ on days 9 and 18 of cultivation, which were over 5- and 82-fold increases in densities when cultured without Maf in Ca_3_(PO_4_)_2_ medium (Table 5). As described above, strain JXJ CY39^T^ would disappear from cultures after co-culture with Maf in BG11 medium for 10 days or more. Thus, these results suggested that Maf could adjust interactions with strain JXJ CY39^T^ when available N and P are limited, and further certified that the interactions between algae and attached bacteria are dynamic and can be initiated and ended in response to environmental change.

### 3.7 Inhibition of attached bacteria by Maf metabolites

Plate-based antibacterial assays (Supplementary Figure S7) revealed that Maf extracts exhibited obvious inhibitory activities on strains JXJ CY 31, 37, and 39, in addition to weak (strains JXJ CY 16 and 28) or no inhibitory activities on other attached bacterial strains. Fraction IV represented the fat-soluble component and did not exhibit inhibitory activity against any of the nine attached bacteria. Fraction II exhibited the strongest inhibitory activity, followed by fraction I. Fraction III contained MC-LR at a concentration of 4 μg per disk and exhibited no or very weak inhibitory activities on any of the nine attached bacteria.

Co-culture analyses (Table 3) revealed that the cell densities of some strains increased at 5 days of cultivation and then decreased at day 10 of cultivation. The reason for this difference was likely because antibacterial compounds secreted by Maf did not achieve concentrations high enough to inhibit bacterial growth after 5 days of cultivation, but did after 10 days of cultivation. However, the cell densities of strains JXJ CY 05, 11, 16, 18, 28, and 57 significantly decreased (*p* < 0.01) after day 10 of cultivation and even day 5 of cultivation, despite that Maf extracts exhibited no or very weak inhibitory activities on these bacteria grown on plates. It is possible that the synthesis of specific antibacterial compounds required the induction of these bacteria or alternatively that some antibacterial compounds lost their antibacterial activities during chemical extraction and isolation.

Healthy macroalgae can control their attached bacteria to avoid excessive bacterial growth and further competition for nutrients (Kouzuma and Watanabe, 2015). Specifically, healthy *M. aeruginosa* can apparently control their attached bacteria to avoid competition for nutrients (Zhang et al., 2016b,c; Xiao et al., 2022a,b,c). Likewise, extracts from *M. aeruginosa* exhibited inhibitory activities on some of their attached bacteria (Casamatta and Wickstrom, 2000) and many other bacterial taxa including *Escherichia coli* (Ostensvik et al., 1998; Valdor and Aboal, 2007), *Streptoverticillium* (Valdor and Aboal, 2007), *Bacillus subtilis*, *B. cereus*, and *Aeromonas hydrophila* (Ostensvik et al., 1998). Microcystins including MC-LR, MC-RR, and MC-YR were also evaluated for antibacterial activity. Ostensvik et al. (1998) observed that MCs did not exhibit any inhibitory activities against *E. coli*, *Bacillus subtilis*, *B. cereus*, and *Aeromonas hydrophila* at a concentration of 1–8 μg mL^−1^, while Valdor and Aboal (2007) observed that MC-YR inhibited *Streptoverticillium* at a 12.5 μg mL^−1^, while MC-RR and MC-LR inhibited *Streptoverticillium* at a 25 μg mL^−1^, and MC-LR inhibited *E. coli* at a 5 μg mL^−1^. Nevertheless, it has remained unclear whether MCs are the specific chemicals by which Maf control their attached bacteria.

MC-LR accounts for over 57% of the MCs produced in *M. aeruginosa* laboratory cultures (Liu et al., 2012) and 45.5–99.8% of the MCs in HAB-impacted natural waters (Vasconcelos et al., 1996). The results of this study showed that E-MC-LR, I-MC-LR, and total MC-LR concentrations were 0.017–0.37, 0.44–1.25, and 0.46–1.36 μg mL^−1^, respectively. Thus, the concentrations of E-MC-LR and even the total MCs from both environmental samples and laboratory culture samples are a small fraction of the concentrations used in previous studies. Consequently, MCs may not be the specific chemicals secreted by Maf that control their attached bacteria. These results were further verified with antibacterial assays (Supplementary Figure S7). Fraction III contained MC-LR at a concentration of 4 μg per disk and exhibited no or very weak inhibitory activities on bacterial strains, while fractions I and II, and especially fraction II, exerted stronger and more broad-spectrum antibacterial activity. Consequently, fraction II likely contains one of the main chemicals that Maf uses to specifically control their attached bacteria.

Co-culture experiments (Table 3) revealed that strain JXJ CY 39^T^ disappeared when co-cultured with Maf in BG11 medium. However, the cellular density of strain JXJ CY 39^T^ on day 18 of cultivation in Ca_3_(PO_4_)_2_ medium was 200% of that on day 9 of cultivation (Table 5). Thus, the concentrations of antibacterial components in Maf cultures did not increase with cultivation time although chl-*a* concentrations increased by 56.2%. Only enough strain JXJ CY 39^T^ biomass can provide Maf with enough available P in Ca_3_(PO_4_)_2_ medium, probably partially explaining why the cell density of strain JXJ CY 39^T^ increased after 18 days of cultivation. Thus, Maf was apparently able to adjust the synthesis and secretion of specific antibacterial components based on their nutritional requirements. And this further proved that the interactions between algae and attached bacteria are dynamic and can be initiated and ended in response to environmental change.

### 3.8 Influence of strain JXJ CY 39^T^ on the growth of non-culturable Maf-attached bacteria

**Figure 3:**
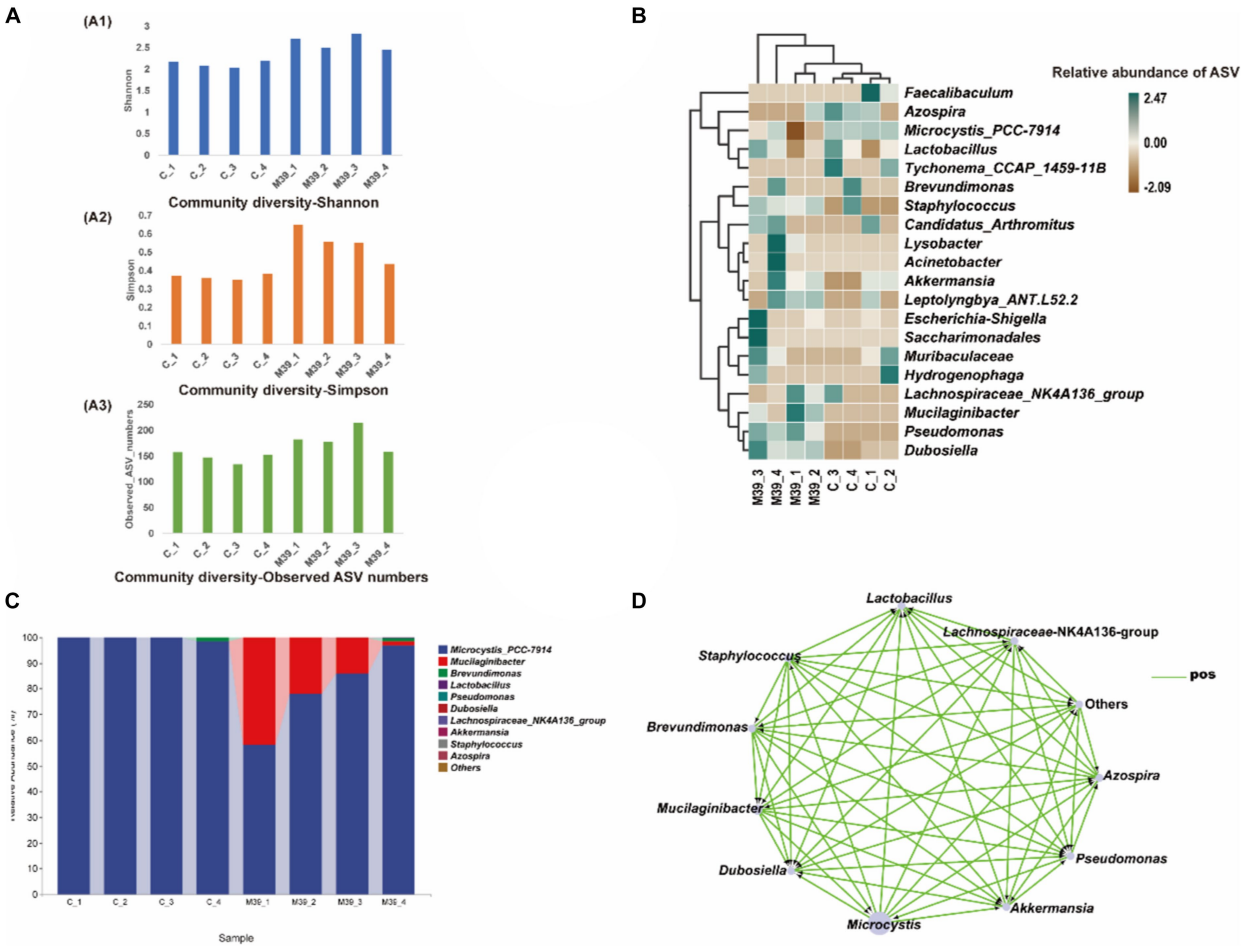
Analysis of bacterial community in samples of purified Maf co-cultured with and without strain JXJ CY 39^T^ on days 5, 10, 15, and 35. **(A)** Amplicon sequence variant (ASV) richness and diversity indices of culture communities assessed with Shannon diversity, Simpson diversity, and total ASV number indices. **(B)** Heatmap analysis of enriched bacterial taxa in these culture samples. **(C)** The relative abundances of the bacterial community at the genus taxon level in purified Maf co-cultured with or without strain JXJ CY 39^T^ on days 5, 10, 15, and 35. The relative abundances of the most abundant genera are shown. **(D)** Co-occurrence network visualization of dominant genera in the communities. Each node represents a dominant genus and each edge represents two correlated genera based on absolute Spearman’s correlations >0.5 and with a *p* < 0.05. The edge length is based on Bray-Curtis distances and the node size reflects the relative abundances of genera. C_1, C_2, C_3, and C_4 indicate cultures of Maf without JXJ CY 39^T^ collected on days 5, 10, 15, and 35 of cultivation, respectively. M39_1, M39_2, M39_3, and M39_4 indicate co-cultures of JXJ CY 39^T^ and Maf collected on days 5, 10, 15, and 35 of cultivation, respectively.

No bacteria grew on ISP2 plates after incubation at 28.0°C for 7 days, indicating that strain JXJ CY 39^T^ in the samples had died, and the control cultures remained axenic during the experiments. Community compositional analyses of eight samples of purified Maf co-cultured with strain JXJ CY 39^T^ and purified Maf cultured without strain JXJ CY 39^T^ from days 5, 10, 15, and 35 yielded 820,153 high-quality, partial 16S rRNA gene sequences without singletons, representing 379 amplicon sequence variants (ASVs). Each sample only contained a few unique ASVs (Supplementary Figure S8). Bacterial intra-sample (alpha) diversity was estimated by rarefaction analysis (Supplementary Figure S9) and by calculating the Shannon and Simpson diversity indices, in addition to the observed number of ASVs (Figure 3A). Rarefaction analyses with the Shannon index indicated that the sequencing efforts recovered nearly all diversity expected in these samples. The community diversity of purified Maf co-cultured with strain JXJ CY 39^T^ was higher than that of purified Maf cultured without strain JXJ CY 39^T^.

ASVs were taxonomically classified using the latest version of the Ribosomal Database Project (RDP) database to assess bacterial taxonomic compositional changes across time in cultures of Maf with and without strain JXJ CY 39^T^. Differences between culture conditions were evident. In the control groups, there were 159, 150, 134, 154 ASVs detected in purified Maf cultured without strain JXJ CY 39^T^ at days 5, 10, 15, 35 of the culture periods, respectively. While in the experimental groups, higher number of ASVs were detected in bacterial communities, with 183, 178, 217 and 157 ASVs detected in purified Maf co-cultured with strain JXJ CY 39^T^ on day 5, 10, 15, 35, respectively. A total of 9 phyla, 22 orders, and 28 genera were identified in culture samples of purified Maf co-cultured with strain JXJ CY 39^T^, while 7 phyla, 20 orders, and 27 genera were identified in culture samples of Maf cultured without strain JXJ CY 39^T^. The relative abundances of Maf cultured without strain JXJ CY 39^T^ were over 98.35% in all samples. Bacteria belonging to several phyla were also detected including *Proteobacteria*, *Bacteroidetes*, *Actinobacteria*, *Firmicutes*, and *Verrucomicrobia* on day 5 of cultivation; *Proteobacteria*, *Bacteroidetes*, *Actinobacteria*, *Firmicutes*, *Verrucomicrobia*, and *Deinococcus-Thermus* on day 10 of cultivation; *Proteobacteria*, *Bacteroidetes*, and *Firmicutes* on day 15 of cultivation; and *Proteobacteria*, *Firmicutes*, *Bacteroidetes*, and *Actinobacteria* on day 35 of cultivation. The relative abundances these phyla ranged between 0.0028–0.05%, except for *Proteobacteria* on day 35 of cultivation, that exhibited relative abundances of 1.59%. Thus, the relative abundances of non-culturable attached bacteria of Maf changed with cultivation time, while the *Proteobacteria*, *Bacteroidetes*, and *Firmicutes,* and especially *Proteobacteria,* were the most common phyla associated with *Microcystis* and were maintained at relatively high abundances. Specifically, on day 5 and 10 of the culture periods, the relative abundance of ASVs belonging to the genus *Dubosiella* was 0.003%, whereas on days 15 and 35 of the culture periods, it dropped to 0%. Similarly, the abundance of ASVs belonging to the genus *Akkermansia* decreased from 0.007% on days 5, and 10 of culture to 0% on days 15, and 35 of culture. On the other hand, the relative abundance of ASVs belonging to the genus *Lactobacillus* was absent (0%) on day 5 of culture, increased to 0.011% on day 10 of culture, 0.026% on day 15 of culture, and decreased to 0.012% on day 35 of the culture period.

The relative abundances of Maf co-cultured with strain JXJ CY 39^T^ were 58.20, 77.94, 85.96, and 96.87% on days 5, 10, 15, and 35 of cultivation, respectively. The relative abundances of strain JXJ CY 39^T^ co-cultured with Maf were 41.72, 21.94, 13.85, and 1.59% on days 5, 10, 15, and 35 of cultivation, respectively. Thus, the relative abundances of Maf increased with cultivation time, in contrast to those of strain JXJ CY 39^T^. The relative abundances of strain JXJ CY 39^T^ quickly decreased, indicating that its fraction of the community decreased with culture time. In addition, many other bacteria from various phyla were detected in the co-culture samples. *Proteobacteria*, *Firmicutes*, and *Verrucomicrobia* were detected on day 5 of cultivation, while *Actinobacteria* appeared on day 10 of cultivation along with the three abovementioned phyla. The *Epsilonbacteraeota*, and *Patescibacteria* likewise appeared on day 15 of cultivation in addition to the four above phyla, while *Deinococcus-Thermus* appeared on day 35 of cultivation alongside concomitant disappearances of *Epsilonbacteraeota* and *Patescibacteria*. The relative abundances of these phyla ranged between 0.0017–0.075%, except for *Proteobacteria* on day 35 of cultivation that exhibited a relative abundance of 1.45%. Notably, the *Epsilonbacteraeota* and *Patescibacteria* were detected in co-cultures of Maf and strain JXJ CY 39^T^, unlike Maf cultured without strain JXJ CY 39^T^.

Linear discriminant analysis (LDA) of effect size (LEfSe) analysis can be used to identify significant enrichment of bacteria among samples (Segata et al., 2011). A cladogram was constructed for LEfSe analysis (Figure 3B) that showed the most differentially abundant bacterial taxa (with default logarithmic, LDA, values >3.0) related to the two groups of samples. The bacterial taxa enriched in culture samples of purified Maf co-cultured with strain JXJ CY 39^T^ were *Mucilaginibacter*, *Dubosiella*, and *Pseudomonas* (Figure 3B). As expected, *Microcystis* was most abundant in culture samples of purified Maf cultured without JXJ CY 39^T^. A total of 16 genera were detected only in co-cultures of purified Maf and strain JXJ CY 39^T^, including *Acidovorax*, *Acinetobacter*, *Aquabacterium*, *Arenimonas*, *Bacteroides*, *Bifidobacterium*, *Bosea*, *Chujaibacter*, *Helicobacter*, *Jeotgalicoccus*, *Lysobacter*, *Oscillibacter*, *Pseudomonas*, *Sphingomonas*, *Sporosarcina*, and *Truepera*. In addition, a total of 13 genera were found only in the control groups, including *Alloprevotella*, *Azonexus*, *Blautia*, *Bradyrhizobium*, *Cellulomonas*, *Devosia*, *Enterorhabdus*, *Faecalibaculum*, *Lawsonella*, *Muribaculum*, *Quadrisphaera*, *Romboutsia*, and *Thermus*. The relative abundances of the 11 most abundant genera across all culture samples were specifically investigated (Figure 3C). *Brevundimonas* was detected in co-culture samples of Maf and strain JXJ CY 39^T^ at day 15 (0.004%), and their relative abundances increased at day 35 (1.401%). In contrast, *Brevundimonas* was not detected in the samples of purified Maf cultured without JXJ CY 39^T^ until day 35 (1.577%). In addition, the relative abundances of *Dubosiella* in co-cultures of purified Maf and strain JXJ CY 39^T^ (0.0098, 0.0111, 0.0182, and 0.0087%; on days 5, 10, 15, and 35, respectively) were all higher than in cultures without JXJ CY 39^T^ (0.0028, 0.0031, 0, and 0%, respectively).

Co-occurrence network analysis was used to identify correlations between genera among samples (Figure 3D). Specifically, the relative abundances of the 11 most dominant genera in cultures with purified Maf co-cultured with and without strain JXJ CY 39^T^ were compared at days 5, 10, 15, and 35 of cultivation. *Mucilaginibacter* (e.g., like strain JXJ CY 39^T^), *Brevundimonas,* and *Pseudomonas* relative abundances were positively associated with Maf growth and Maf was positively correlated to the relative abundances of *Akkermansia*, *Azospira*, *Dubosiella*, *Lactobacillus*, and *Staphylococcus*. Consistently, the chl-*a* concentrations in some co-cultures of purified Maf and strain JXJ CY 39^T^ were higher than control values in BG11 medium. In addition, strains JXJ CY 11 and 57 belonged to the genus *Pseudomonas*. On day 5 of cultivation, the I-MC-LR concentration of BCS with strain JXJ CY 11 were 9.7% higher (*p* < 0.05) than that of the control. On day 10 of cultivation, the I-MC-LR concentration of BCS with strain JXJ CY 57 were 19.6% higher (*p* < 0.01) than that of the control. Thus, *Mucilaginibacter* and *Pseudomonas* may positively influence the growth of Maf, consistent with the co-occurrence network analyses. Thus, co-culture with strain JXJ CY 39^T^ may also influence the growth of other attached bacteria of Maf.

### 3.9 Description of *Mucilaginibacter lacusdianchii* sp. nov.

*Mucilaginibacter lacusdianchii* sp. nov. (la.cus.di.a’n.chii L. gen. n. *lacus*, of a lake; N.L. gen. n. *dianchii*, of Dianchi; N.L. gen. n. *lacusdianchii*, of Dianchi lake).

Strain JXJ CY 39^T^ is aerobic, Gram-negative, rod-shaped (0.7–1.0 × 0.9–2.0 μm) and grows well on ISP2 medium. The pH, NaCl content (*w*/*v*), and temperature ranges for growth are 4.0–11.0, 0–3.0%, and 5–38°C, respectively, with optimal growth at pH 7.0–8.0, 0% NaCl, and 28.0°C, respectively. The strain is positive for catalase, oxidase, nitrate reduction, hydrolysis of starch, Tween 40 and 80, and negative for Tween 20 hydrolysis. The major cellular fatty acids were iso-C_15:0_ and C_16:1_ω7c/_16:1_ω6c. The predominant respiratory quinone is MK-7. The polar lipids are phosphatidylethanolamine (PE), unidentified aminophosphoglycolipid (APGL), unidentified aminoglycolipids (AGL), an unidentified phospholipid (PL), and unidentified polar lipids (L1-3).

The type strain, JXJ CY 39^T^ (= KCTC 72617^T^ = CGMCC 1.17449^T^), was isolated from the culture biomass of *Microcystis aeruginosa* FACHB-905 collected from Lake Dianchi in the Yunnan province of southwestern China. The genome has a G + C content of 42.1%. The GenBank accession numbers for the 16S rRNA gene sequence and draft genome sequence of strain JXJ CY 39^T^ are MT674523 and WSRW00000000, respectively.

## 4 Conclusion

A novel species (type strain JXJ CY 39^T^) of the genus *Mucilaginibacter* was discovered from the phycosphere of Maf and we established *Mucilaginibacter lacusdianchii* sp. nov. based on the polyphasic taxonomic study. The interplay between the Maf-associated bacteria and their host is intricate and fluctuates with time. Additional research is necessary to determine if manipulating these microbial interactions could serve as a viable strategy for controlling Harmful Algal Blooms (HABs).

## Data availability statement

The datasets presented in this study can be found in online repositories. The names of the repository/repositories and accession number(s) can be found in the article/Supplementary material.

## Author contributions

YX: Data curation, Investigation, Validation, Writing – original draft, Writing – review & editing. MD: Investigation, Validation, Writing – original draft, Writing – review & editing. YD: Data curation, Validation, Visualization, Writing – original draft, Writing – review & editing. QD: Investigation, Validation, Data curation, Resources, Writing – review & editing. XW: Investigation, Validation, Data curation, Writing – review & editing. YY: Investigation, Validation, Writing – review & editing. BZ: Conceptualization, Funding acquisition, Supervision, Validation, Writing – original draft, Writing – review & editing. Y-QZ: Conceptualization, Formal analysis, Funding acquisition, Methodology, Supervision, Validation, Writing – original draft, Writing – review & editing.

## Footnotes

^1^ https://lpsn.dsmz.de/genus/mucilaginibacter

^2^ https://www.ncbi.nlm.nih.gov/genome/

^3^ https://github.com/zdf1987/EasyCGTree4
