# Supplementary material for: Modulation of growth, microcystin production, and algal-bacterial interactions of the bloom-forming algae Microcystis aeruginosa by a novel bacterium recovered from its phycosphere
Source: Front Microbiol. 2024 Mar 1;15:1295696. doi: 10.3389/fmicb.2024.1295696 (PMC10940515; doi:10.3389/fmicb.2024.1295696)
Supplement: Supplementary file 1 [file Data_Sheet_1.pdf]

Modulation of growth, microcystin production, and algal-bacterial interactions of the bloom-forming algae *Microcystis aeruginosa* by a novel bacterial isolate recovered from its phycosphere

Yao Xiao[a‡], Mijia Du[a‡], Yang Deng[b‡], Qinglin Deng[a], Xin Wang[a], Yi-Wen Yang[a], Binghuo Zhang[a*], Yu-Qin Zhang[b*]

[a] *College of Pharmacy and Life Science, Jiujiang University, Jiujiang, 332000, China*

[b]*Institute of Medicinal Biotechnology, Chinese Academy of Medical Sciences & Peking Union Medical College, Beijing 100050, China*

[*]**Corresponding authors:**

E-*mail address*: yzhang@imb.pumc.edu.cn (Y. Zhang);

binghuozh@126.com (B. Zhang)

[‡]Yao Xiao, Mijia Du and Yang Deng shared the position of first author.

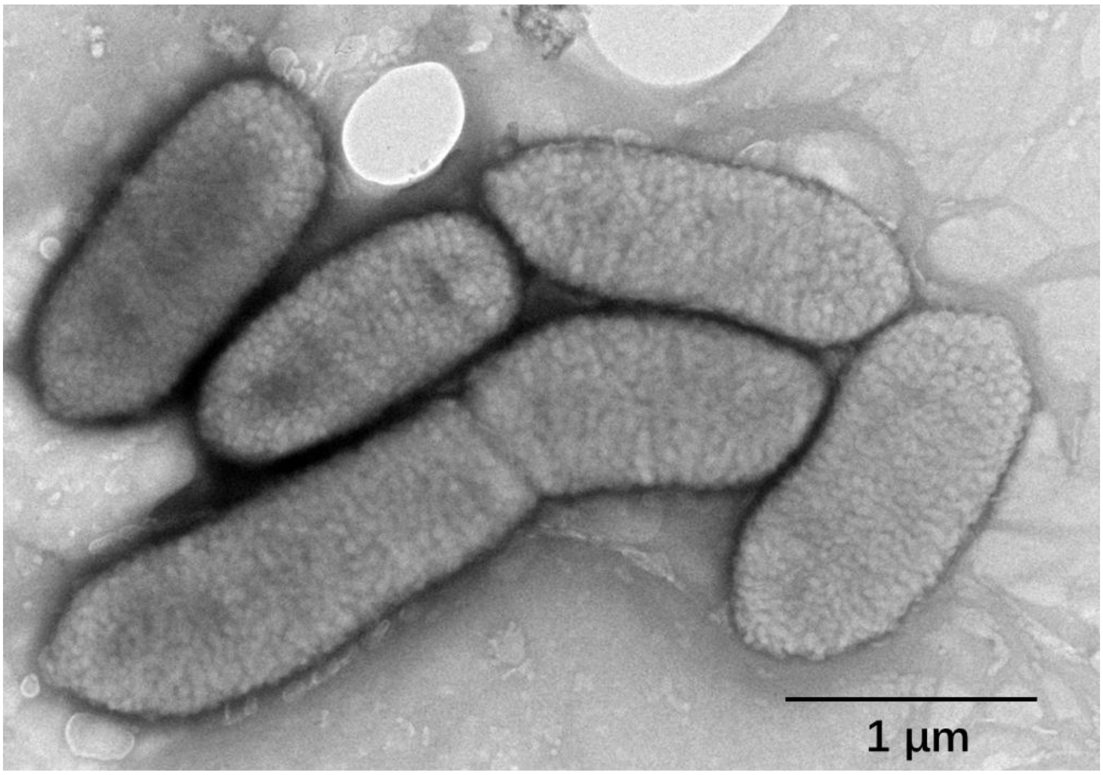

Fig. S1. Transmission electron micrograph of strain JXJ CY 39^T.

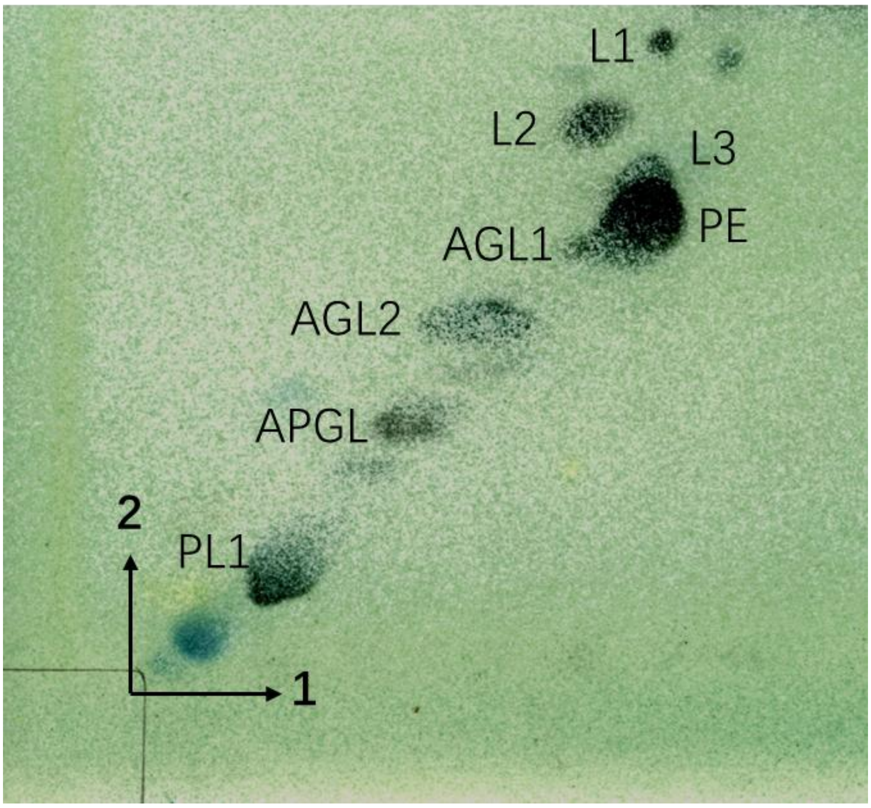

Fig. S2. The polar lipids of strain JXJ CY 39^T revealed by two-dimensional thin layer chromatography. PE, phosphatidylethanolamine; APGL, unidentified aminophosphoglycolipid; AGL, unidentified aminoglycolipids; PL, unidentified phospholipid; L1-3, unidentified polar lipids.

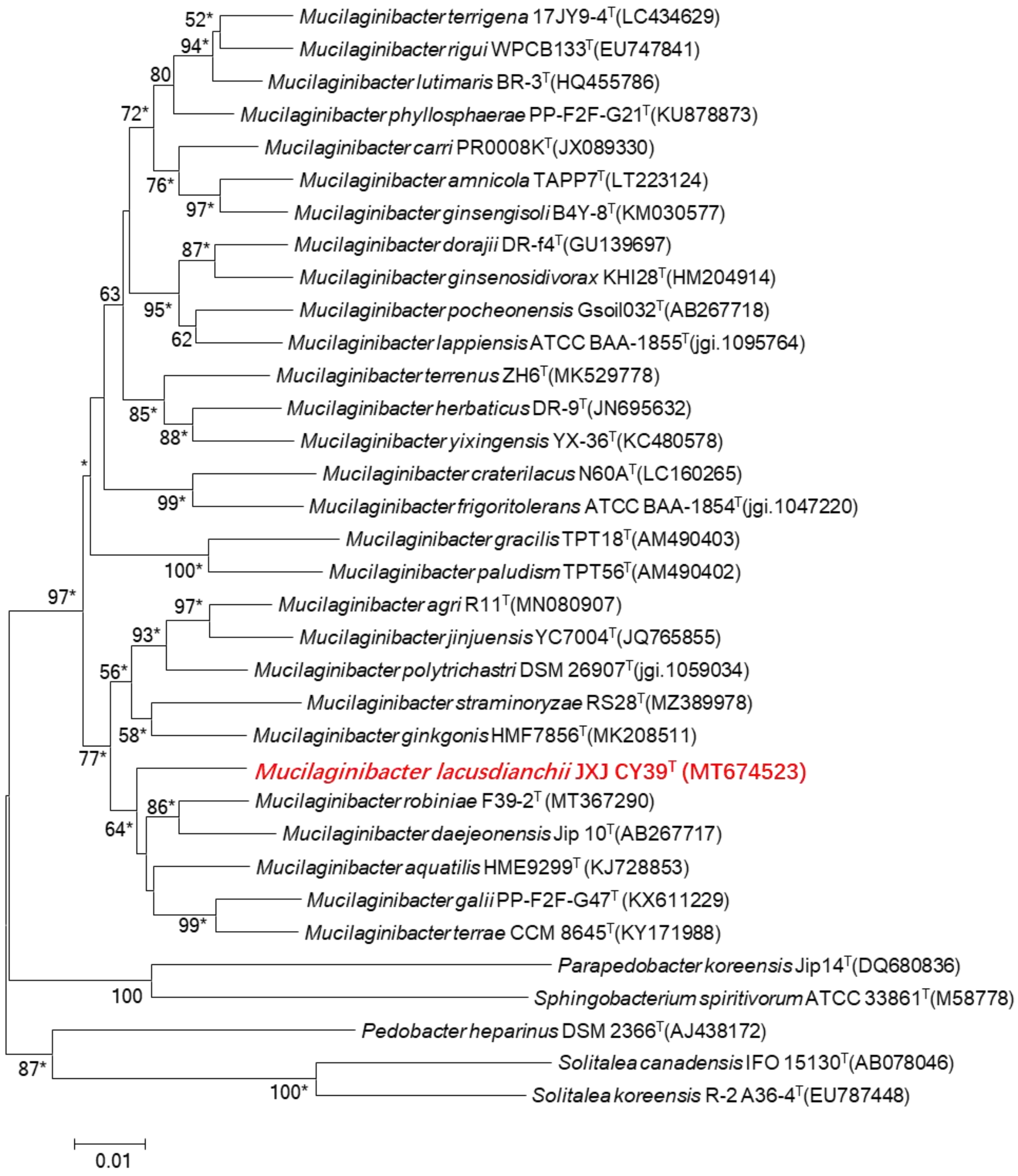

Fig. S3. Neighbor-joining phylogenetic tree based on 16S rRNA gene sequences of strain JXJ CY 39^T and the most closely related species of the genus *Mucilaginibacter*. Bootstrap values ≥50% are shown on branch nodes and are based on 1,000 replicates. Asterisks indicate that the corresponding nodes were conserved in trees generated with Maximum-Likelihood and maximum-parsimony algorithms. Bar, 0.01 changes per nucleotide position.

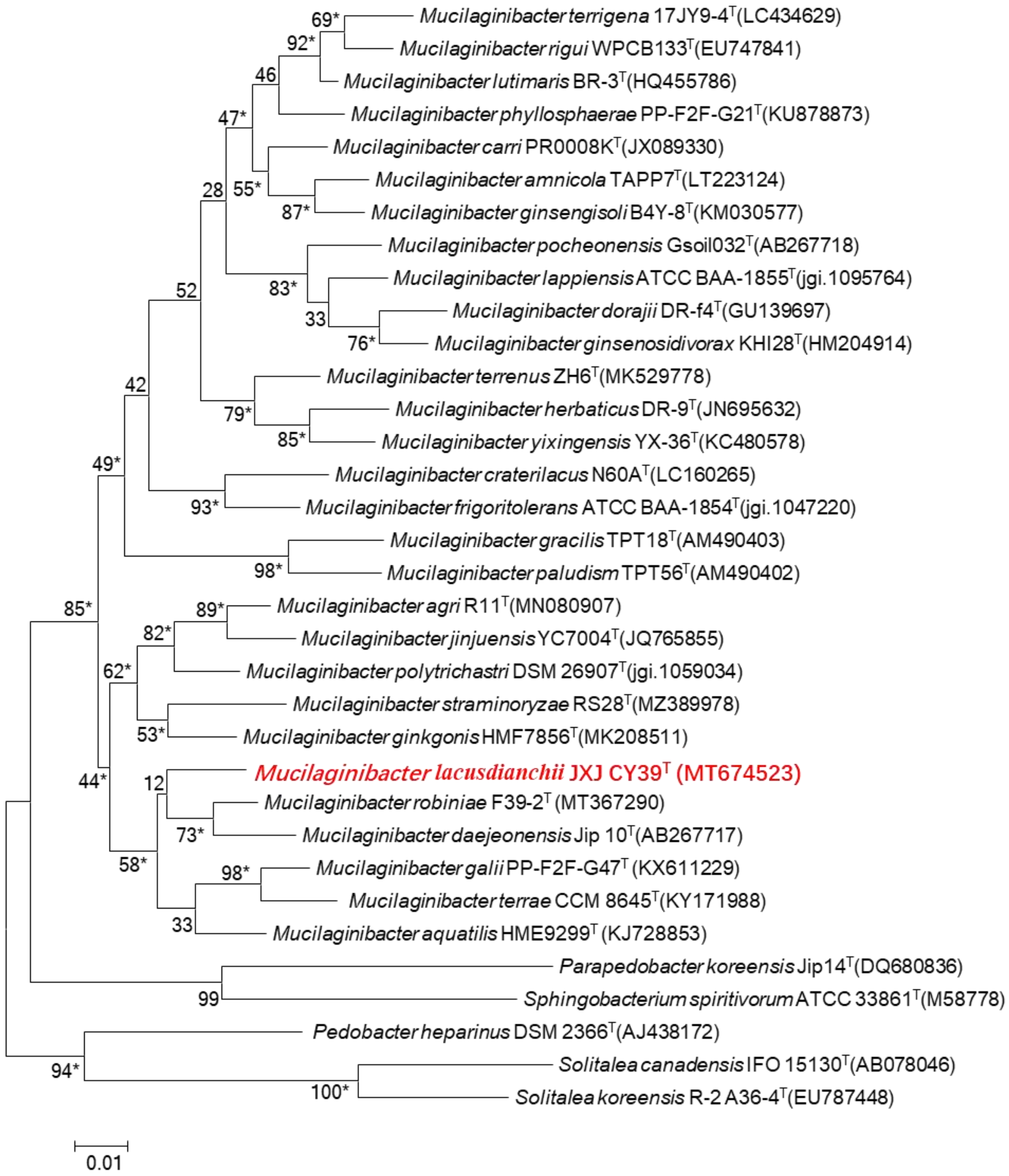

Fig. S4. Maximum-Likelihood phylogenetic tree based on 16S rRNA gene sequences of strain JXJ CY 39^{T} and its closest related species in the genus *Mucilaginibacter*. * indicates clades that were conserved in neighbor-joining, maximum-likelihood, and maximum-parsimony trees. Bar, 0.01 changes per nucleotide position.

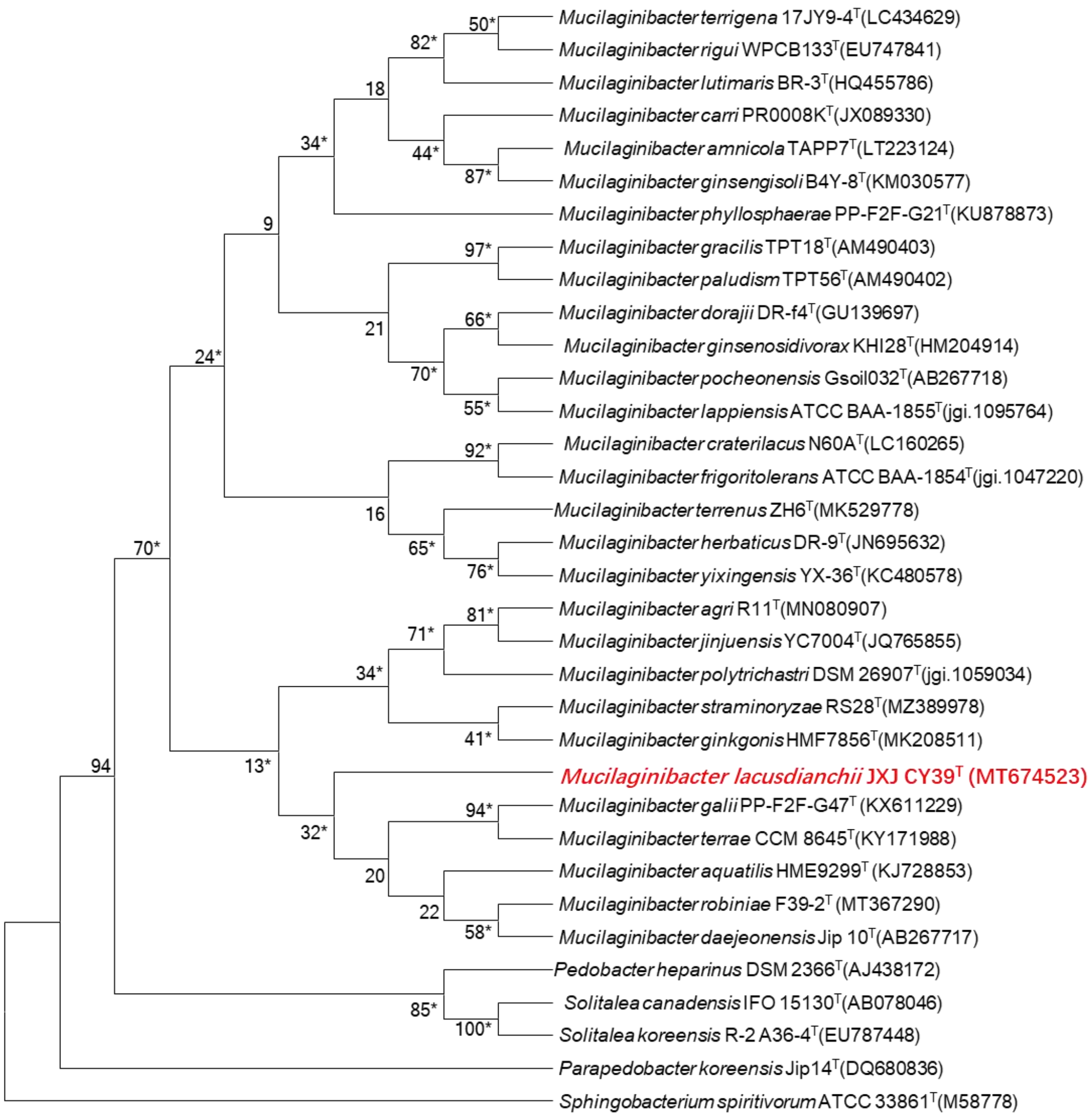

Fig. S5. Maximum-parsimony phylogenetic tree based on 16S rRNA gene sequences of strain JXJ CY 39^{T} and its closest related species in the genus *Mucilaginibacter*. * indicates clades that were conserved in neighbor-joining, Maximum-Likelihood, and maximum-parsimony trees.

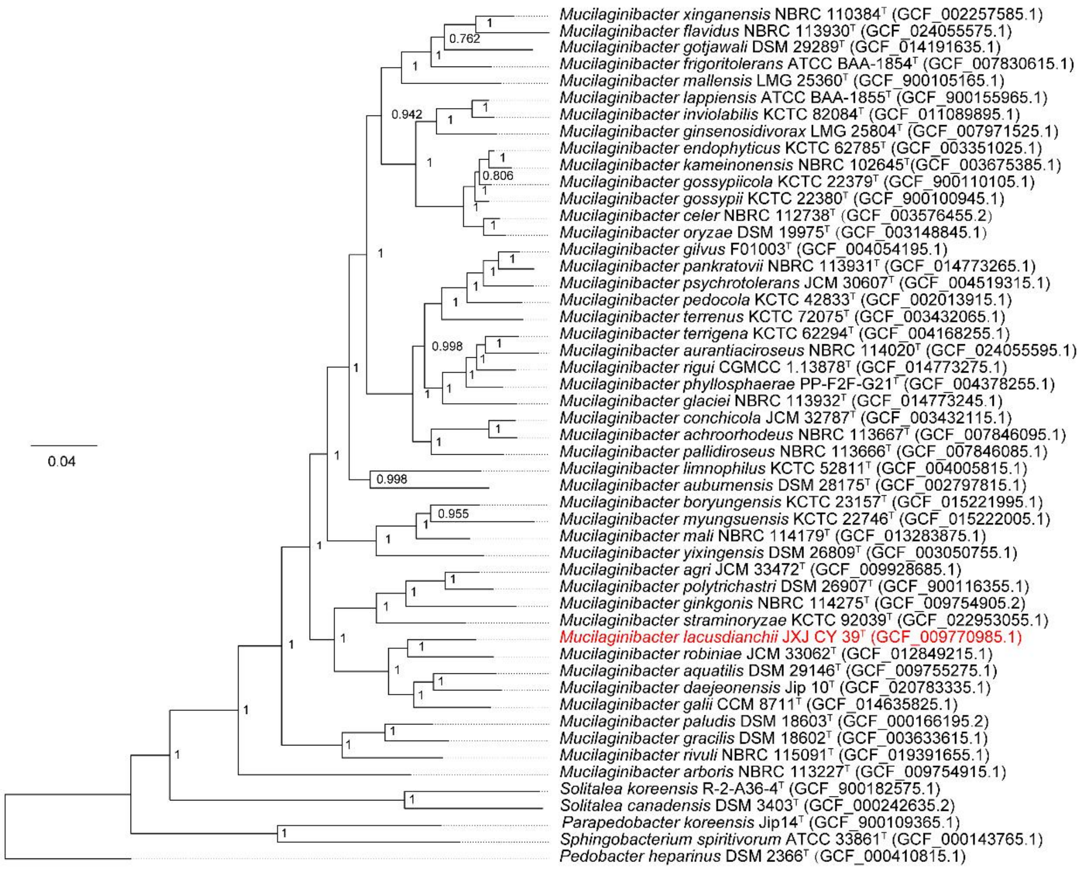

Fig. S6. Maximum-Likelihood core gene phylogenomic tree. Strain JXJ CY 39^T is highlighted in red. Bootstrap values are shown at the branch nodes. RefSeq assembly accession numbers are indicated in brackets for reference genomes. Bar, 0.04 substitutions per nucleotide position.

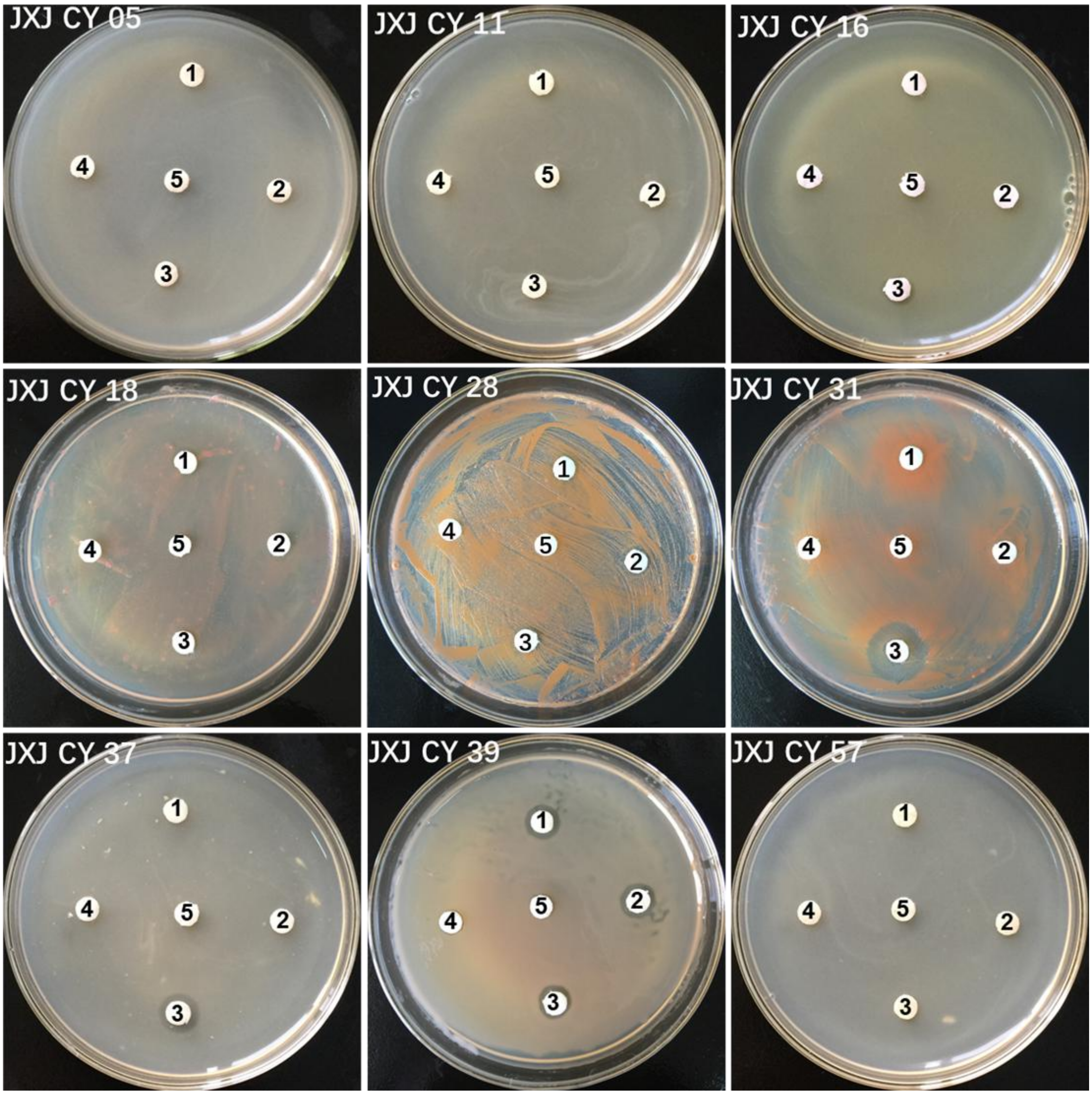

Fig. S7. Inhibitory activity of extracts from Maf on nine attached bacterial strains. 1, 2, 3, 4, and 5 indicate the total extract from Maf, in addition to fractions I, II, III, and IV, respectively. Only fraction III contained MC-LR.

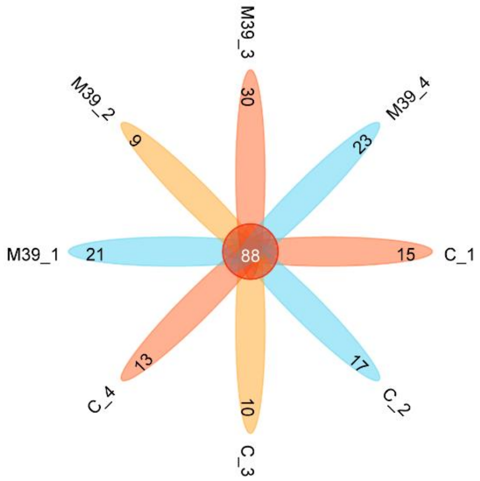

Fig. S8. Venn diagram showing shared and unique amplicons sequence variants (ASVs) among communities from Maf with or without co-culture including strain JXJ CY 39^T at days 5, 10, 15, 35. C_1, C_2, C_3, and C_4 indicate cultures of Maf without JXJ CY 39^T collected on days 5, 10, 15, and 35 of cultivation, respectively. M39_1, M39_2, M39_3, and M39_4 indicate co-cultures of JXJ CY 39^T and Maf collected on days 5, 10, 15, and 35 of cultivation, respectively. The two numbers in the ovals represent the number of ASVs unique to each sample (number at the end of the oval) and the number of shared ASVs (in the center circle).

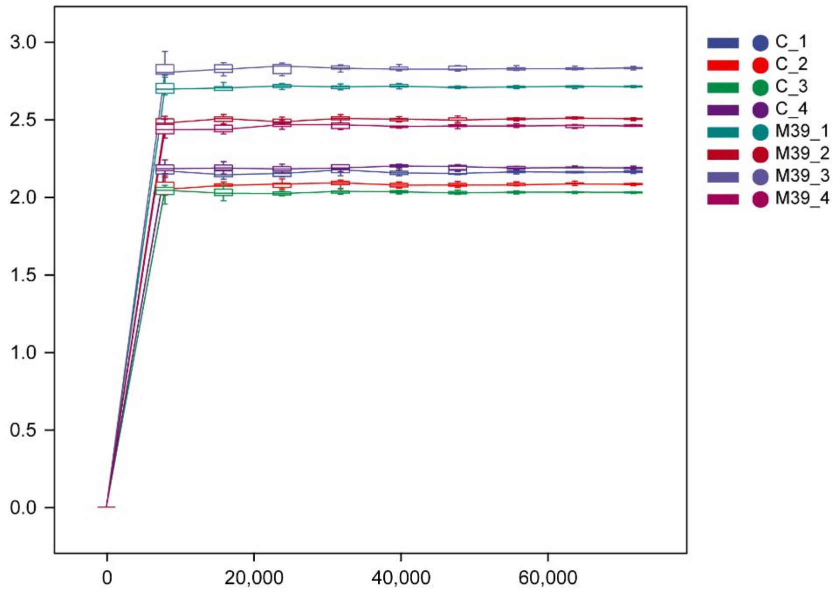

Fig. S9. Rarefaction curves based on the Shannon index of diversity for eight culture samples of Maf with or without co-culture of strain JXJ CY 39^T at days 5, 10, 15, and 35. C_1, C_2, C_3, and C_4 indicate cultures of Maf without JXJ CY 39^T collected on days 5, 10, 15, and 35 of cultivation, respectively. M39_1, M39_2, M39_3, and M39_4 indicate co-cultures of JXJ CY 39^T and Maf collected on days 5, 10, 15, and 35 of cultivation, respectively.

Table S1. Cellular fatty acid profile of strain JXJ CY 39^T and the closely related *M. aquatilis* HME9299^T reference strain.

Fatty acid	1	2	3
$C_{14:0}$	0.7	1.8	0.6
$C_{16:0}$	1.1	3.8	2.3
$C_{15:1}\omega6c$	-	-	1.2
$C_{16:1}\omega5c$	4.1	6.0	10.3
$C_{17:1}\omega8c$	0.1	-	1.2
iso-$C_{15:0}$	45.0	43.2	30.0
iso-$C_{17:0}$	0.3	2.2	-
anteiso-$C_{15:0}$	0.3	-	0.9
$C_{15:0}$ 2-OH	-	-	0.9
$C_{16:0}$ 2-OH	0.1	-	0.9
$C_{16:0}$ 3-OH	0.7	-	1.0
$C_{18:0}$ 3-OH	0.1	-	-
iso-$C_{15:0}$ 3-OH	2.7	1.8	1.3
iso-$C_{17:0}$ 3-OH	6.1	9.6	5.9
$C_{16:1}\omega7c/_{16:1}\omega6c$	30.3	23.5	36.2
Iso-$C_{17:1}$ I/anteiso-$C_{17:1}$ B	1.4	1.0	0.9
$C_{18:1}\omega7c$	0.5	-	-
iso-$C_{17:1}$ $\omega9c$ and/or 10-methyl $C_{16:0}$	2.5	4.6	3.6

1, JXJ CY 39^T; 2, *M. robiniae* F39- 2^T (Won et al., 2022); 3, *M. aquatilis* HME9299^T (Kang et al., 2021). -, no fatty acids were detected.

Table S2. Potentially important genes or gene clusters of strain JXJ CY 39^T related to adaptation to Maf ecology.

Level	GO ID	GO term	Ontology	Number of genes	Gene list
2	GO:0002376	Immune system process	Biological process	4	PROKKA_03601, PROKKA_02744, PROKKA_01575, PROKKA_00029
3	GO:0002520	Immune system development	Biological process	1	PROKKA_01575
3	GO:0002682	Regulation of immune system process	Biological process	1	PROKKA_01575
3	GO:0006955	Immune response	Biological process	2	PROKKA_03601, PROKKA_00029
3	GO:0044110	Growth involved in symbiotic interaction	Biological process	4	PROKKA_03263, PROKKA_01068, PROKKA_01529, PROKKA_02791
4	GO:0052173	Response to defenses of other organisms involved in symbiotic interaction	Biological process	4	PROKKA_02808, PROKKA_01235, PROKKA_03263, PROKKA_01192
5	GO:0051807	Evasion or tolerance of defense response of other organisms involved in symbiotic interaction	Biological process	2	PROKKA_01235, PROKKA_01192
5	GO:0051832	Avoidance of defenses of other organisms involved in symbiotic interaction	Biological process	2	PROKKA_01235, PROKKA_01192
5	GO:0052564	Response to immune response of other organisms involved in symbiotic interaction	Biological process	4	PROKKA_02808, PROKKA_01235, PROKKA_03263, PROKKA_01192
6	GO:0051805	Evasion or tolerance of immune response of other organisms involved in symbiotic interaction	Biological process	2	PROKKA_01235, PROKKA_01192
6	GO:0051834	Evasion or tolerance of defenses of other organisms involved in symbiotic interaction	Biological process	2	PROKKA_01235, PROKKA_01192
6	GO:0052550	Response to defense-related reactive oxygen species production by other organisms involved in symbiotic interaction	Biological process	1	PROKKA_01192
6	GO:0052551	Response to defense-related nitric oxide production by other organisms involved in symbiotic interaction	Biological process	1	PROKKA_01192
6	GO:0052572	Response to host immune response	Biological process	4	PROKKA_02808, PROKKA_01235, PROKKA_03263, PROKKA_01192
7	GO:0020012	Evasion or tolerance of host immune response	Biological process	2	PROKKA_01235, PROKKA_01192
7	GO:0051810	Active evasion of immune response of other organisms involved in symbiotic interaction	Biological process	1	PROKKA_01235
7	GO:0052376	Evasion or tolerance by organism of nitric oxide produced by other organisms involved in symbiotic interaction	Biological process	1	PROKKA_01192
7	GO:0052385	Evasion or tolerance by organism of reactive oxygen species produced by other organisms involved in symbiotic interaction	Biological process	1	PROKKA_01192
8	GO:0042783	Aactive evasion of host immune response	Biological process	1	PROKKA_01235

Table S3. Potentially important genes and gene clusters related to nutrient exchange between strain JXJ CY 39^T and Maf.

Level	GO ID	GO Term	Ontology	Number of genes	Gene list
3	GO:0043190	ATP-binding cassette (ABC) transporter complex	Cellular component	4	PROKKA_03816, PROKKA_00661, PROKKA_00176, PROKKA_04461
3	GO:0015627	Type II protein secretion system complex	Cellular component	1	PROKKA_04344
3	GO:0030256	Type I protein secretion system complex	Cellular component	1	PROKKA_00039
5	GO:0009306	Protein secretion	Biological process	8	PROKKA_04344, PROKKA_00837, PROKKA_04334, PROKKA_00039, PROKKA_01195, PROKKA_00823, PROKKA_04343, PROKKA_00836
6	GO:0015628	Protein secretion by the type II secretion system	Biological process	2	PROKKA_04344, PROKKA_04334
6	GO:0030253	Protein secretion by the type I secretion system	Biological process	1	PROKKA_00039
4	GO:0009341	Beta-galactosidase complex	Cellular component	3	PROKKA_02275, PROKKA_03137, PROKKA_03940
6	GO:0009044	Xylan 1,4-beta-xylosidase activity	Molecular function	6	PROKKA_02227, PROKKA_02228, PROKKA_03588, PROKKA_02220, PROKKA_02217, PROKKA_02031
6	GO:0015923	Mannosidase activity	Molecular function	7	PROKKA_02560, PROKKA_01079, PROKKA_04030, PROKKA_04025, PROKKA_02449, PROKKA_03089, PROKKA_03938
6	GO:0015925	Galactosidase activity	Molecular function	14	PROKKA_04044, PROKKA_02284, PROKKA_02216, PROKKA_00533, PROKKA_03124, PROKKA_02275, PROKKA_02283, PROKKA_03121, PROKKA_03929, PROKKA_03092, PROKKA_03137, PROKKA_03940, PROKKA_04031, PROKKA_01641
6	GO:0015926	Glucosidase activity	Molecular function	14	PROKKA_04372, PROKKA_02297, PROKKA_04374, PROKKA_02519, PROKKA_04452, PROKKA_04424, PROKKA_03743, PROKKA_04411, PROKKA_04376, PROKKA_00563, PROKKA_02530, PROKKA_01654, PROKKA_03448, PROKKA_02528
6	GO:0015928	Fucosidase activity	Molecular function	5	PROKKA_03088, PROKKA_03094, PROKKA_01780, PROKKA_02518, PROKKA_03120
6	GO:0015929	Hexosaminidase activity	Molecular function	3	PROKKA_03093, PROKKA_03099, PROKKA_02321
6	GO:0016160	Amylase activity	Molecular function	4	PROKKA_03742, PROKKA_02491, PROKKA_03529, PROKKA_02492
6	GO:0046556	Alpha-N-arabinofuranosidase activity	Molecular function	7	PROKKA_01461, PROKKA_04403, PROKKA_02228, PROKKA_02220, PROKKA_02215, PROKKA_02217, PROKKA_02031
6	GO:0046559	Alpha-glucuronidase activity	Molecular function	3	PROKKA_02114, PROKKA_02107, PROKKA_02229
6	GO:0051669	Fructan beta-fructosidase activity	Molecular function	1	PROKKA_02410
6	GO:0080176	Xyloglucan 1,6-alpha-xylosidase activity	Molecular function	1	PROKKA_03049
7	GO:0004556	Alpha-amylase activity	Molecular function	4	PROKKA_03742, PROKKA_02491, PROKKA_03529, PROKKA_02492
7	GO:0004557	Alpha-galactosidase activity	Molecular function	5	PROKKA_02284, PROKKA_02283, PROKKA_02216, PROKKA_00533, PROKKA_04031
7	GO:0004558	Alpha-glucosidase activity	Molecular function	4	PROKKA_00563, PROKKA_01654, PROKKA_03743, PROKKA_02297
7	GO:0004573	Mannosyl-oligosaccharide glucosidase activity	Molecular function	1	PROKKA_04452
7	GO:0008422	Beta-glucosidase activity	Molecular function	9	PROKKA_02530, PROKKA_04372, PROKKA_04374, PROKKA_02519, PROKKA_03448, PROKKA_04424, PROKKA_02528, PROKKA_04411, PROKKA_04376
7	GO:0004559	Alpha-mannosidase activity	Molecular function	3	PROKKA_04030, PROKKA_04025, PROKKA_03938
7	GO:0004560	Alpha-L-fucosidase activity	Molecular function	5	PROKKA_03088, PROKKA_03094, PROKKA_01780, PROKKA_02518, PROKKA_03120
7	GO:0004567	Beta-mannosidase activity	Molecular function	4	PROKKA_02449, PROKKA_02560, PROKKA_01079, PROKKA_03089
7	GO:0004573	Mannosyl-oligosaccharide glucosidase activity	Molecular function	1	PROKKA_04452
8	GO:0008496	Mannan endo-1,6-alpha-mannosidase activity	Molecular function	2	PROKKA_04030, PROKKA_04025
8	GO:0015924	Mannosyl-oligosaccharide mannosidase activity	Molecular function	1	PROKKA_03938
8	GO:0016985	Mannan endo-1,4-beta-mannosidase activity	Molecular function	1	PROKKA_02449

8	GO:0008706	6-phospho-beta-glucosidase activity	Molecular function	1	PROKKA_04424
8	GO:0032450	Maltose alpha-glucosidase activity	Molecular function	4	PROKKA_00563, PROKKA_01654, PROKKA_03743, PROKKA_02297
8	GO:0042973	Glucan endo-1,3-beta-D-glucosidase activity	Molecular function	1	PROKKA_02530
8	GO:0046557	Glucan endo-1,6-beta-glucosidase activity	Molecular function	2	PROKKA_04376, PROKKA_04374
5	GO:0016052	Carbohydrate catabolic process	Biological process	76	PROKKA_03597, PROKKA_03529, PROKKA_03196, PROKKA_01341, PROKKA_02114, PROKKA_02037, PROKKA_01010, PROKKA_03870, PROKKA_02108, PROKKA_00693, PROKKA_03746, PROKKA_01673, PROKKA_04030, PROKKA_04025, PROKKA_01533, PROKKA_01741, PROKKA_02011, PROKKA_02111, PROKKA_02528, PROKKA_01575, PROKKA_04408, PROKKA_04101, PROKKA_04404, PROKKA_02031, PROKKA_02129, PROKKA_01577, PROKKA_03074, PROKKA_02229, PROKKA_02463, PROKKA_00941, PROKKA_02719, PROKKA_03966, PROKKA_01134, PROKKA_01667, PROKKA_03579, PROKKA_02220, PROKKA_03131, PROKKA_03476, PROKKA_04405, PROKKA_02228, PROKKA_00384, PROKKA_03099, PROKKA_03988, PROKKA_01534, PROKKA_01767, PROKKA_02130, PROKKA_03117, PROKKA_04409, PROKKA_01928, PROKKA_00633, PROKKA_00927, PROKKA_03448, PROKKA_04406, PROKKA_02215, PROKKA_00787, PROKKA_01745, PROKKA_02758, PROKKA_02131, PROKKA_04373, PROKKA_02106, PROKKA_02219, PROKKA_00637, PROKKA_02218, PROKKA_02217, PROKKA_02163, PROKKA_02224, PROKKA_02107, PROKKA_03696, PROKKA_04100, PROKKA_03109, PROKKA_03588, PROKKA_03885, PROKKA_00860, PROKKA_00329, PROKKA_04023, PROKKA_02759
5	GO:0043470	Regulation of carbohydrate catabolic process	Biological process	1	PROKKA_01575
6	GO:0044724	Single-organism carbohydrate catabolic process	Biological process	49	PROKKA_03476, PROKKA_03597, PROKKA_04405, PROKKA_03196, PROKKA_01341, PROKKA_01010, PROKKA_03988, PROKKA_03870, PROKKA_01534, PROKKA_02108, PROKKA_01767, PROKKA_00693, PROKKA_03746, PROKKA_02130, PROKKA_03117, PROKKA_01928, PROKKA_01673, PROKKA_01533, PROKKA_00633, PROKKA_01741, PROKKA_02011, PROKKA_02111, PROKKA_00927, PROKKA_03448, PROKKA_02528, PROKKA_04406, PROKKA_01575, PROKKA_00787, PROKKA_04101, PROKKA_04404, PROKKA_01745, PROKKA_02758, PROKKA_02129, PROKKA_02131, PROKKA_01577, PROKKA_04373, PROKKA_00637, PROKKA_02163, PROKKA_00941, PROKKA_02719, PROKKA_04100, PROKKA_03109, PROKKA_01134, PROKKA_01667, PROKKA_03885, PROKKA_00860, PROKKA_00329, PROKKA_04023, PROKKA_02759
8	GO:0006596	Polyamine biosynthetic process	Biological process	3	PROKKA_03795, PROKKA_02479, PROKKA_02480
6	GO:0010252	Auxin homeostasis	Biological process	3	PROKKA_02309, PROKKA_01270, PROKKA_00854
6	GO:0080030	Methyl indole-3-acetate esterase activity	Molecular function	4	PROKKA_01913, PROKKA_02833, PROKKA_04428, PROKKA_01272
3	GO:0009349	Riboflavin synthase complex	Cellular component	1	PROKKA_01090
3	GO:0051183	Vitamin transporter activity	Molecular function	4	PROKKA_02524, PROKKA_02427, PROKKA_04047, PROKKA_03776
4	GO:0004076	Biotin synthase activity	Molecular function	1	PROKKA_03841
4	GO:0051180	Vitamin transport	Biological process	1	PROKKA_02931
4	GO:0015235	Cobalamin transporter activity	Molecular function	4	PROKKA_02524, PROKKA_02427, PROKKA_04047, PROKKA_03776
5	GO:0015889	Cobalamin transport	Biological process	1	PROKKA_02931
7	GO:0009236	Cobalamin biosynthetic process	Biological process	3	PROKKA_02483, PROKKA_01102, PROKKA_03580
5	GO:0004746	Riboflavin synthase activity	Molecular function	1	PROKKA_01877
5	GO:0009110	Vitamin biosynthetic process	Biological process	35	PROKKA_00851, PROKKA_03801, PROKKA_00950, PROKKA_00738, PROKKA_02928, PROKKA_03580, PROKKA_03450, PROKKA_02483, PROKKA_01203, PROKKA_02645, PROKKA_02978, PROKKA_02712, PROKKA_01364, PROKKA_03884, PROKKA_00324,

					PROKKA_03155, PROKKA_03906, PROKKA_01987, PROKKA_03802, PROKKA_01877, PROKKA_04277, PROKKA_01204, PROKKA_03280, PROKKA_03841, PROKKA_01143, PROKKA_01131, PROKKA_03836, PROKKA_00909, PROKKA_01090, PROKKA_04387, PROKKA_03896, PROKKA_00313, PROKKA_01468, PROKKA_01102, PROKKA_01900
6	GO:0004141	Dethiobiotin synthase activity	Molecular function	1	PROKKA_03801
6	GO:0009102	Biotin biosynthetic process	Biological process	6	PROKKA_03896, PROKKA_03801, PROKKA_03906, PROKKA_03841, PROKKA_03802, PROKKA_02645
6	GO:0009229	Thiamine diphosphate biosynthetic process	Biological process	4	PROKKA_01364, PROKKA_03280, PROKKA_00738, PROKKA_04387
6	GO:0042364	Water-soluble vitamin biosynthetic process	Biological process	35	PROKKA_00851, PROKKA_03801, PROKKA_00950, PROKKA_00738, PROKKA_02928, PROKKA_03580, PROKKA_03450, PROKKA_02483, PROKKA_01203, PROKKA_02645, PROKKA_02978, PROKKA_02712, PROKKA_01364, PROKKA_03884, PROKKA_00324, PROKKA_03155, PROKKA_03906, PROKKA_01987, PROKKA_03802, PROKKA_01877, PROKKA_04277, PROKKA_01204, PROKKA_03280, PROKKA_03841, PROKKA_01143, PROKKA_01131, PROKKA_03836, PROKKA_00909, PROKKA_01090, PROKKA_04387, PROKKA_03896, PROKKA_00313, PROKKA_01468, PROKKA_01102, PROKKA_01900
7	GO:0009228	Thiamine biosynthetic process	Biological process	5	PROKKA_01364, PROKKA_03280, PROKKA_00738, PROKKA_00909, PROKKA_04387
7	GO:0009231	Riboflavin biosynthetic process	Biological process	7	PROKKA_01468, PROKKA_01131, PROKKA_03836, PROKKA_02928, PROKKA_01877, PROKKA_01090, PROKKA_02978
7	GO:0009234	Menaquinone biosynthetic process	Biological process	6	PROKKA_03861, PROKKA_00365, PROKKA_00427, PROKKA_00949, PROKKA_02749, PROKKA_04287
7	GO:0042819	Vitamin B6 biosynthetic process	Biological process	5	PROKKA_03884, PROKKA_03155, PROKKA_01900, PROKKA_01143, PROKKA_04277
6	GO:0016791	Phosphatase activity	Molecular function	44	PROKKA_03242, PROKKA_01067, PROKKA_01466, PROKKA_01614, PROKKA_04192, PROKKA_00083, PROKKA_01959, PROKKA_02489, PROKKA_02312, PROKKA_02877, PROKKA_01543, PROKKA_01078, PROKKA_01847, PROKKA_03150, PROKKA_01539, PROKKA_01303, PROKKA_04402, PROKKA_01750, PROKKA_03860, PROKKA_01180, PROKKA_02675, PROKKA_03307, PROKKA_03461, PROKKA_03609, PROKKA_02390, PROKKA_00216, PROKKA_01084, PROKKA_01944, PROKKA_00561, PROKKA_02510, PROKKA_01607, PROKKA_00217, PROKKA_02844, PROKKA_03264, PROKKA_01863, PROKKA_03916, PROKKA_01389, PROKKA_04449, PROKKA_02156, PROKKA_01860, PROKKA_03937, PROKKA_01515, PROKKA_00800, PROKKA_00151
7	GO:0003993	Acid phosphatase activity	Molecular function	1	PROKKA_02844
7	GO:0004035	Alkaline phosphatase activity	Molecular function	2	PROKKA_01067, PROKKA_01303
4	GO:0015849	Organic acid transport	Biological process	5	PROKKA_00969, PROKKA_00970, PROKKA_02744, PROKKA_00339, PROKKA_01678
5	GO:0005342	Organic acid transmembrane transporter activity	Molecular function	19	PROKKA_02030, PROKKA_01698, PROKKA_01268, PROKKA_02824, PROKKA_00025, PROKKA_03241, PROKKA_02674, PROKKA_01344, PROKKA_02855, PROKKA_03255, PROKKA_03494, PROKKA_02285, PROKKA_00537, PROKKA_03652, PROKKA_01533, PROKKA_02746, PROKKA_02744, PROKKA_01128, PROKKA_03610
5	GO:0016053	Organic acid biosynthetic process	Biological process	185	PROKKA_01138, PROKKA_03862, PROKKA_02012, PROKKA_03686, PROKKA_01964, PROKKA_00924, PROKKA_00653, PROKKA_03490, PROKKA_02712, PROKKA_00357, PROKKA_00324, PROKKA_03189, PROKKA_00890, PROKKA_03154, PROKKA_00987, PROKKA_02810, PROKKA_00806, PROKKA_04073, PROKKA_01692, PROKKA_03865, PROKKA_01961, PROKKA_03994, PROKKA_03979, PROKKA_03079, PROKKA_03126, PROKKA_00952, PROKKA_01956, PROKKA_02485, PROKKA_03364, PROKKA_02962, PROKKA_00608, PROKKA_03896, PROKKA_00551, PROKKA_02777, PROKKA_04086, PROKKA_01815, PROKKA_02861, PROKKA_01958, PROKKA_00215, PROKKA_00660,

					PROKKA_00851, PROKKA_00985, PROKKA_03801, PROKKA_00659, PROKKA_00950, PROKKA_02815, PROKKA_04275, PROKKA_01804, PROKKA_03645, PROKKA_03479, PROKKA_01203, PROKKA_01137, PROKKA_04162, PROKKA_03533, PROKKA_03724, PROKKA_03884, PROKKA_03481, PROKKA_00870, PROKKA_01305, PROKKA_01987, PROKKA_01537, PROKKA_00657, PROKKA_00390, PROKKA_00214, PROKKA_01400, PROKKA_03833, PROKKA_01329, PROKKA_01605, PROKKA_02938, PROKKA_03605, PROKKA_01821, PROKKA_03732, PROKKA_00872, PROKKA_03734, PROKKA_04420, PROKKA_00307, PROKKA_00913, PROKKA_00393, PROKKA_03488, PROKKA_03912, PROKKA_00988, PROKKA_01100, PROKKA_01445, PROKKA_02713, PROKKA_03450, PROKKA_03472, PROKKA_00925, PROKKA_00650, PROKKA_01207, PROKKA_00807, PROKKA_01760, PROKKA_04008, PROKKA_02150, PROKKA_03626, PROKKA_03279, PROKKA_01220, PROKKA_03860, PROKKA_03544, PROKKA_01960, PROKKA_00654, PROKKA_01678, PROKKA_04147, PROKKA_00991, PROKKA_03729, PROKKA_04441, PROKKA_00778, PROKKA_01704, PROKKA_01235, PROKKA_03264, PROKKA_03704, PROKKA_03723, PROKKA_02165, PROKKA_00871, PROKKA_00884, PROKKA_01629, PROKKA_02862, PROKKA_03611, PROKKA_04447, PROKKA_00986, PROKKA_01957, PROKKA_02482, PROKKA_00800, PROKKA_03830, PROKKA_01962, PROKKA_03545, PROKKA_01691, PROKKA_03644, PROKKA_00919, PROKKA_01714, PROKKA_03864, PROKKA_01959, PROKKA_02953, PROKKA_01615, PROKKA_02819, PROKKA_00597, PROKKA_02645, PROKKA_00951, PROKKA_01792, PROKKA_03883, PROKKA_03480, PROKKA_03328, PROKKA_01361, PROKKA_04006, PROKKA_03906, PROKKA_00706, PROKKA_03802, PROKKA_00992, PROKKA_00656, PROKKA_04154, PROKKA_01204, PROKKA_00949, PROKKA_01136, PROKKA_02007, PROKKA_00298, PROKKA_03731, PROKKA_03866, PROKKA_02119, PROKKA_00417, PROKKA_00846, PROKKA_03730, PROKKA_03063, PROKKA_03841, PROKKA_03489, PROKKA_01521, PROKKA_03447, PROKKA_03594, PROKKA_02725, PROKKA_04010, PROKKA_02775, PROKKA_03916, PROKKA_00313, PROKKA_01975, PROKKA_00804, PROKKA_00651, PROKKA_01572, PROKKA_00649, PROKKA_02728, PROKKA_00968, PROKKA_03491, PROKKA_00967, PROKKA_01404, PROKKA_00085, PROKKA_01145, PROKKA_01139, PROKKA_03625
5	GO:0009877	Nodulation	Biological process	11	PROKKA_03041, PROKKA_03040, PROKKA_03230, PROKKA_02245, PROKKA_01732, PROKKA_03613, PROKKA_03323, PROKKA_00984, PROKKA_01402, PROKKA_01733, PROKKA_02948
5	GO:0009399	Nitrogen fixation	Biological process	6	PROKKA_02443, PROKKA_02418, PROKKA_01255, PROKKA_03524, PROKKA_00988, PROKKA_00826
3	GO:0004601	Peroxidase activity	Molecular function	14	PROKKA_00643, PROKKA_01782, PROKKA_03856, PROKKA_01192, PROKKA_00389, PROKKA_03101, PROKKA_01193, PROKKA_03783, PROKKA_00319, PROKKA_01301, PROKKA_04223, PROKKA_03216, PROKKA_00512, PROKKA_03367
4	GO:0004602	Glutathione peroxidase activity	Molecular function	2	PROKKA_00319, PROKKA_01301
4	GO:0016691	Chloride peroxidase activity	Molecular function	1	PROKKA_01782
4	GO:0004096	Catalase activity	Molecular function	3	PROKKA_03783, PROKKA_04223, PROKKA_03367
5	GO:0016117	Carotenoid biosynthetic process	biological_process	7	PROKKA_04080, PROKKA_04077, PROKKA_02807, PROKKA_02810, PROKKA_04084, PROKKA_02806, PROKKA_03668
